# Supplementary material for: Multiplex neurodegeneration proteotoxicity platform reveals DNAJB6 promotes non-toxic FUS condensate gelation and inhibits neurotoxicity
Source: Nat Commun. 2025 Nov 21;16:10285. doi: 10.1038/s41467-025-65178-0 (PMC12638947; doi:10.1038/s41467-025-65178-0)
Supplement: Supplementary file 1 — Supplementary Information [file 41467_2025_65178_MOESM1_ESM.pdf]

Multiplex Neurodegeneration Proteotoxicity Platform Reveals DNAJB6 Promotes non-toxic FUS Condensate Gelation and Inhibits Neurotoxicity

Samuel J. Resnick, Seema Qamar, Pushya Krishna, Vladislav Korobeynikov, Hannes Ausserwoger, Alyssa Miller, Pietro Esposito, Juan A. Varela, Jenny Sheng, Lei Haley Huang, Jonathon Nixon-Abell, Schuyler Melore, Chyi Wei Chung, Nino F. Läubli, Sofia Kapsiani, Xuecong Li, Jingshu Wang, Nancy Zhang, Mahabub Maraj Alam, Alondra S. Burguete, Theresa C Swayne, Yanyan Chen, Ya-Cheng Liao, Neil A. Shneider, Michele Vendruscolo, Tuomas P.J. Knowles, Clemens F. Kaminski, Francesco Simone Ruggeri, Gabriele S. Kaminski Schierle, Peter St George-Hyslop, Alejandro Chavez

**Supplementary Information**

## Supplementary Figures

**Supplementary Figure 1. Detailed development of a multiplexed screening platform. a.** Individual yeast strains containing an integrated DNA barcode are transformed with a construct encoding a protein associated with neurodegeneration or control protein before pooling. **b.** Rescuers are introduced *en masse* through mating and selection. **c.** Mated barcode pools are grown in inducing media in a 96-well plate format before DNA harvesting, NGS, and subsequent analysis.

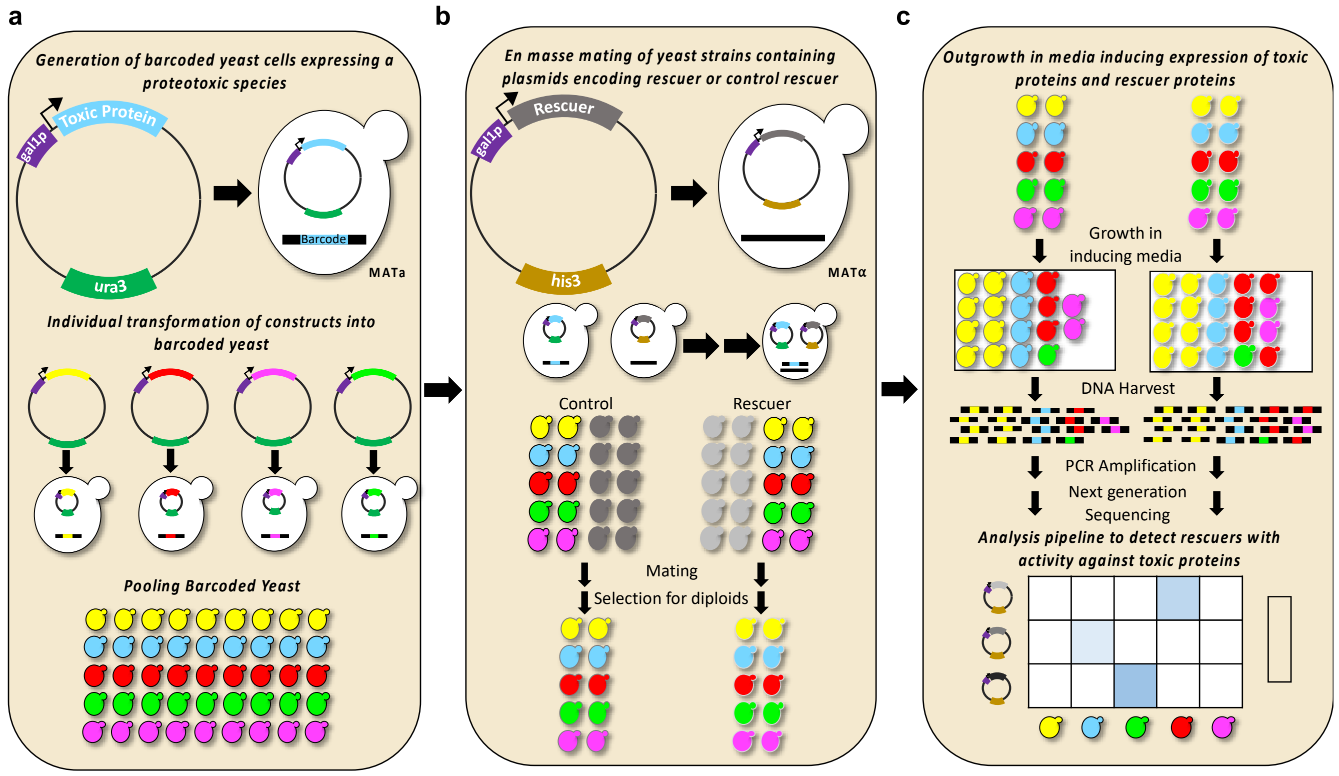

**Supplementary Figure 2.** ***En masse*** **mating of a barcode pool and outgrowth of a mated barcoded pool do not perturb barcode ratios. a.** Example of correlation plot between two separately mated pools that have been selected for diploids, each dot represents a different barcode within the population. **b.** Correlation values for 36 comparisons between the barcode abundance for separately mated pools. **c.** Example of correlation plot between two separately mated pools that have been selected for diploids, and outgrown in inducing media, each dot represents a different barcode within the population. **d.** Correlation values for 36 comparisons between separately mated and outgrown pools.

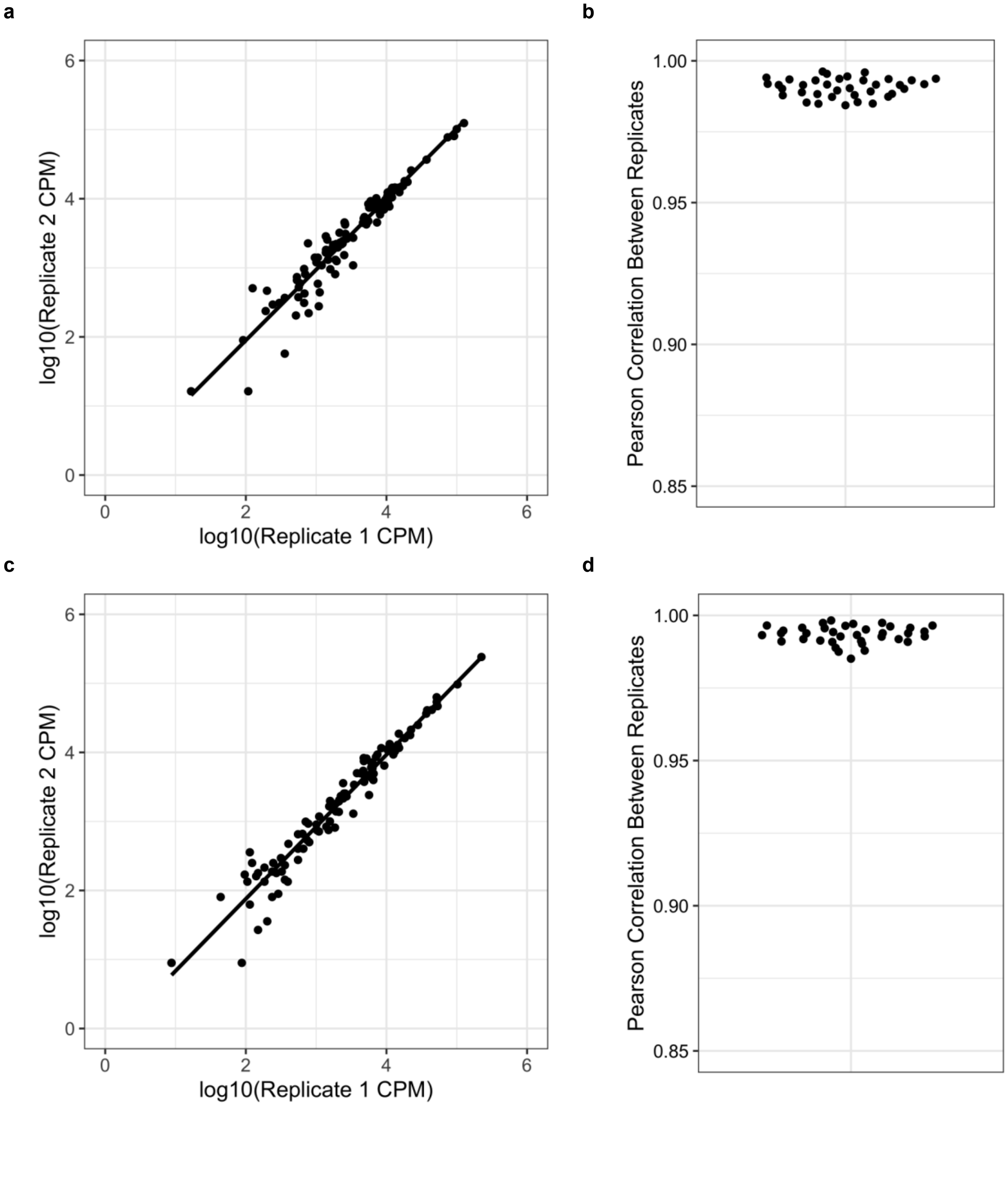

**Supplementary Figure 3. Comparison of three pooling strategies for detecting known interactions**. **a.** Log2 fold change heatmaps for 3 pooling strategies. Previously known interactions that are expected are outlined in purple **b.** Correlations between barcodes after pooled barcoded strains were mated to the same control rescuer, selected for diploids, and grown under inducing condition using each of the three different pooling strategies. **c.** Coefficient of variation vs. relative barcode abundance plot for each of the 3 pooling strategies.

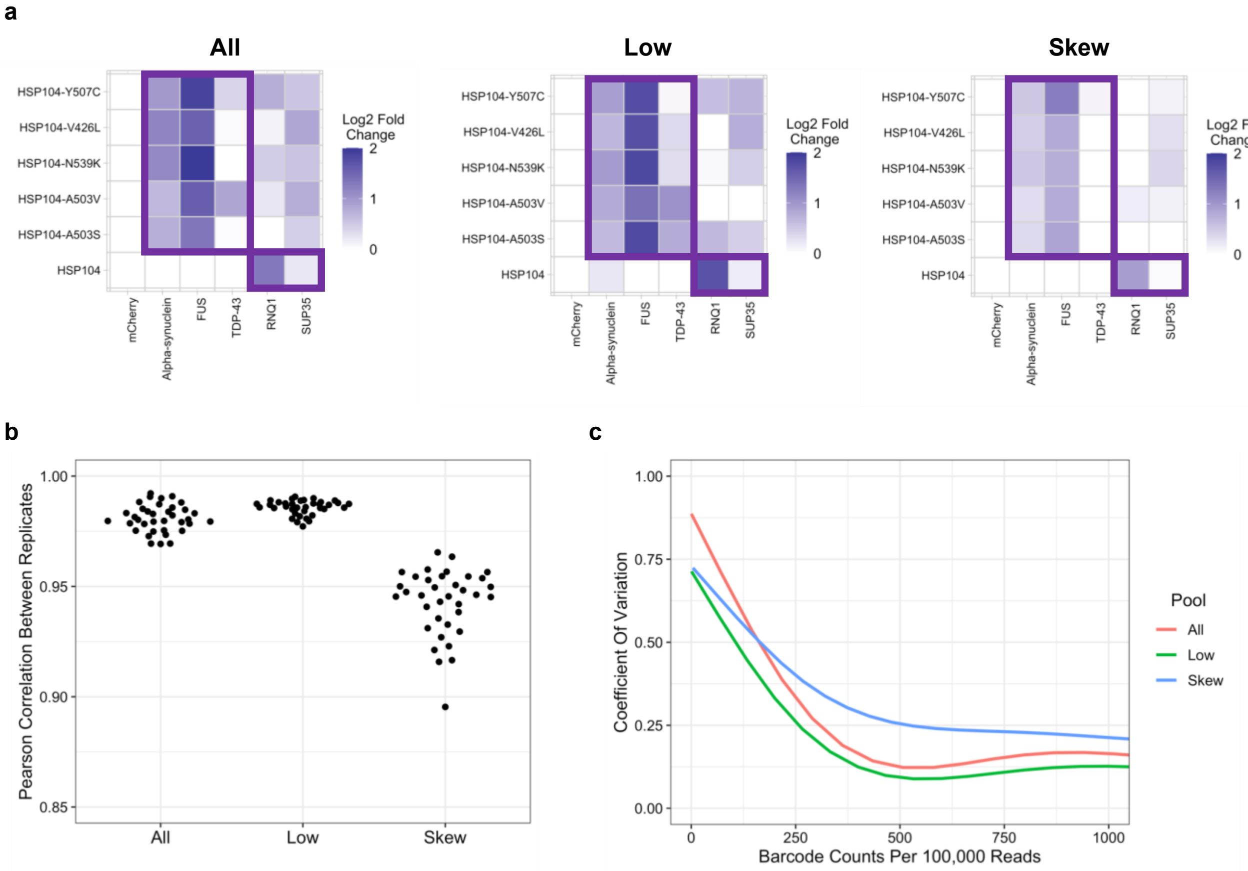

**Supplementary Figure 4. Exploration of biological and technical sources of error for the optimal screening strategy. a.** Correlation between biological replicates (separately mated, selected, outgrown, harvested, and PCR amplified) and technical replicates (same sample of harvested DNA separately PCR amplified). **b.** Coefficient of variation vs. relative barcode abundance plot for biological replicates of pooled DNA-barcoded library mated to an inert rescuer demonstrating the effect of averaging between biological replicates. **c.** Coefficient of variation vs. relative barcode abundance plot for technical replicates of pooled DNA-barcoded library mated to an inert rescuer demonstrating effect of averaging between technical replicates.

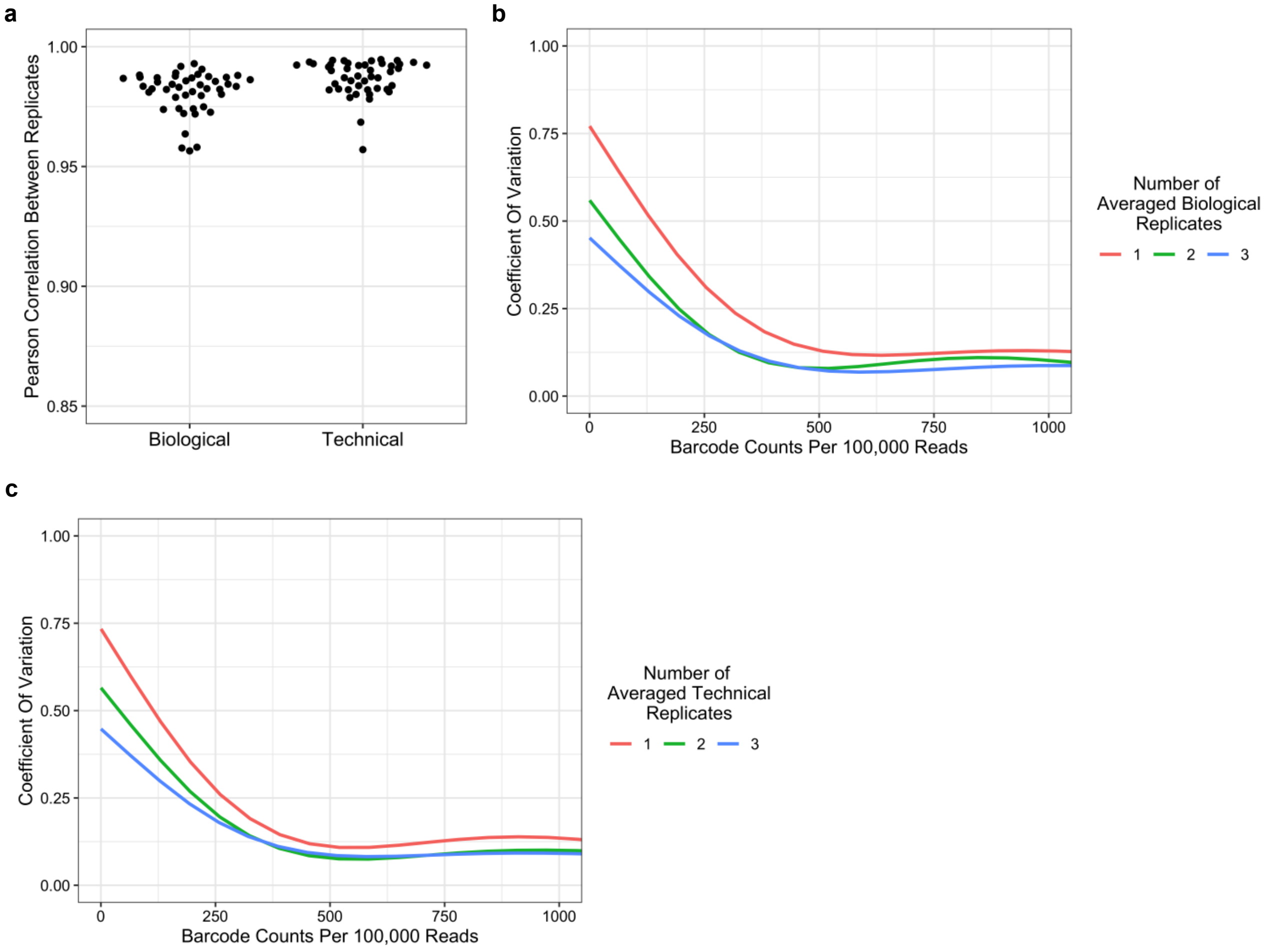

**Supplementary Figure 5. Validation of the optimized multiplexed screening approach using known genetic interactions. a.** Optimized conditions enable detection of positive control interactions in pilot screen **b-g.** Spot assay validation of tested interactions in pilot multiplexed screen.

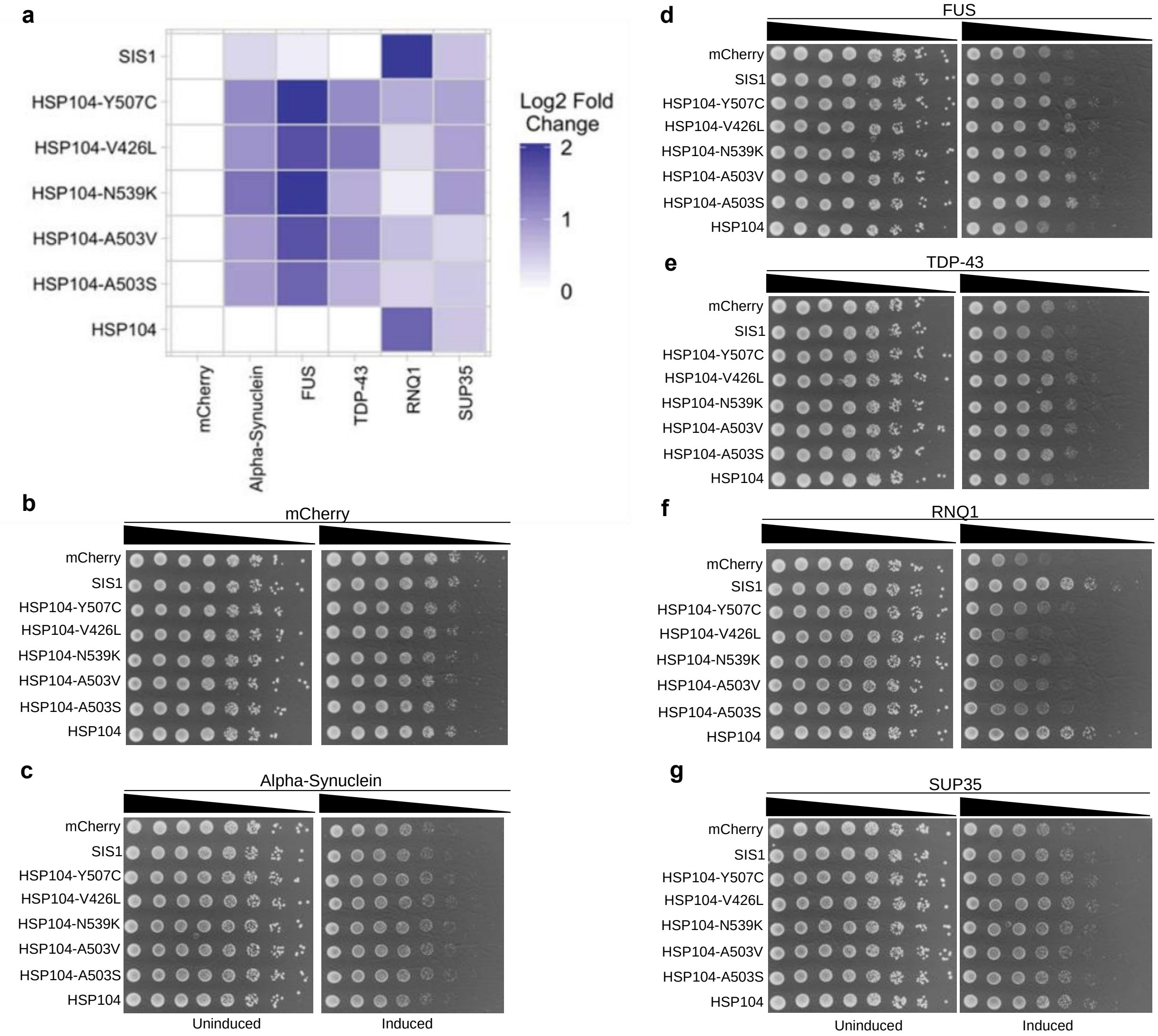

**Supplementary Figure 6. Simulation of how redundant barcoding enhances the ability to reject the null hypothesis.** Fold change required for rejection of null hypothesis simulated using optimized pooling conditions with two biological replicates and two technical replicates.

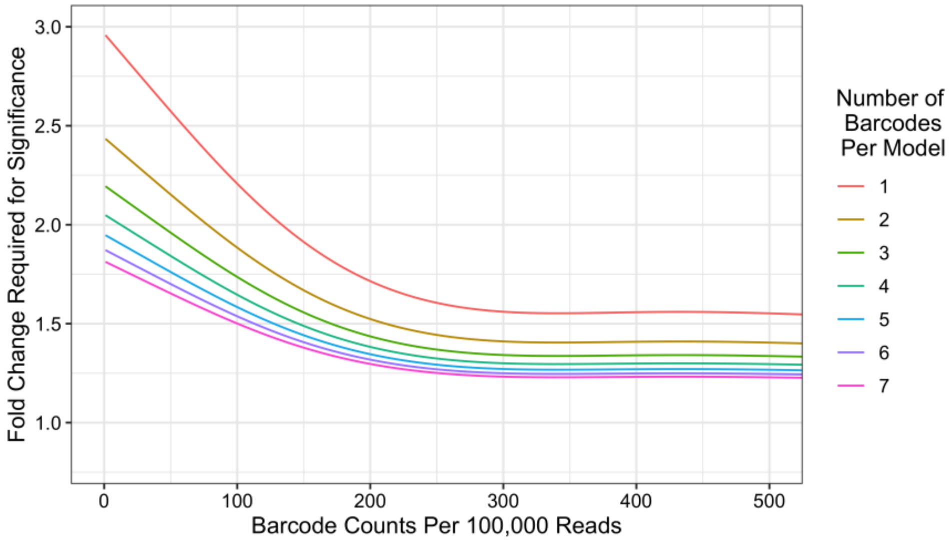

**Supplementary Figure 7. Determination of number of reads required to adequately sample 302-member DNA-barcoded pool**. **a.** Coefficient of variation vs. relative barcode abundance for DNA-barcoded library mated to an inert rescuer at different levels of read subsampling. **b.** Number of individual barcoded strains with less than 10 raw reads at different levels of read subsampling.

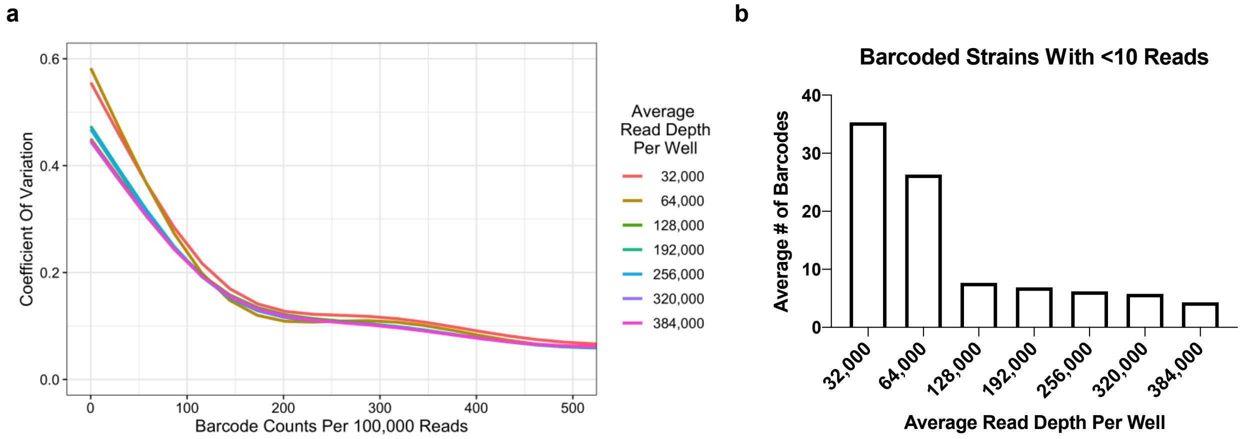

**Supplementary Figure 8. Full yeast chaperone screen. a.** Log2 fold change interactions between all tested yeast chaperones and the models included in the pool. **b.** Significant interactions between yeast chaperones and models included in the pool.

a

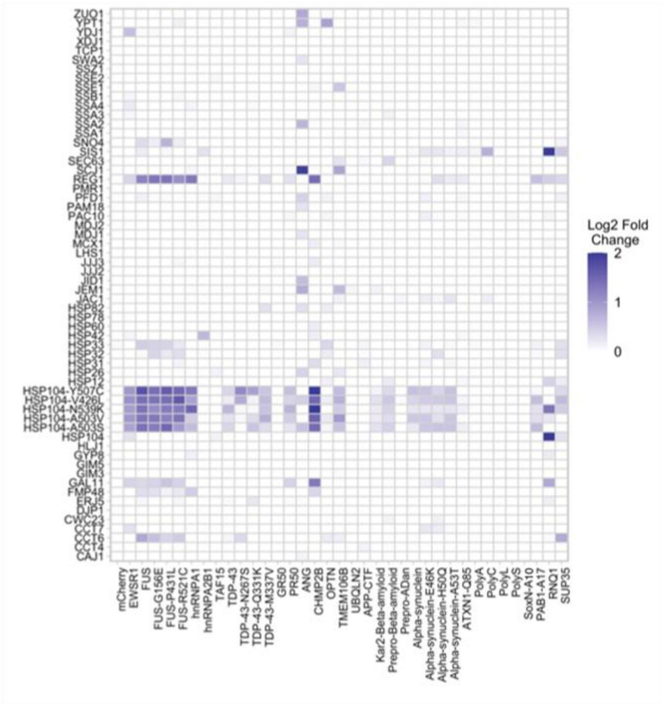


b

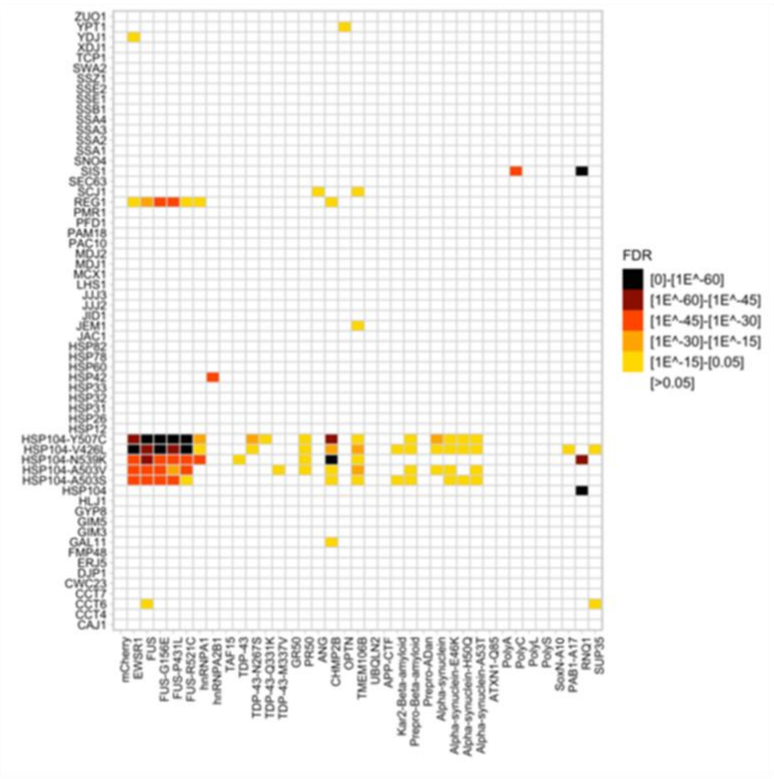

**Supplementary Figure 9. Full human chaperone screen. a.** Log2 fold change interactions between all tested human chaperones and the models included in the pool. **b.** Significant interactions between human chaperones and models included in the pool.

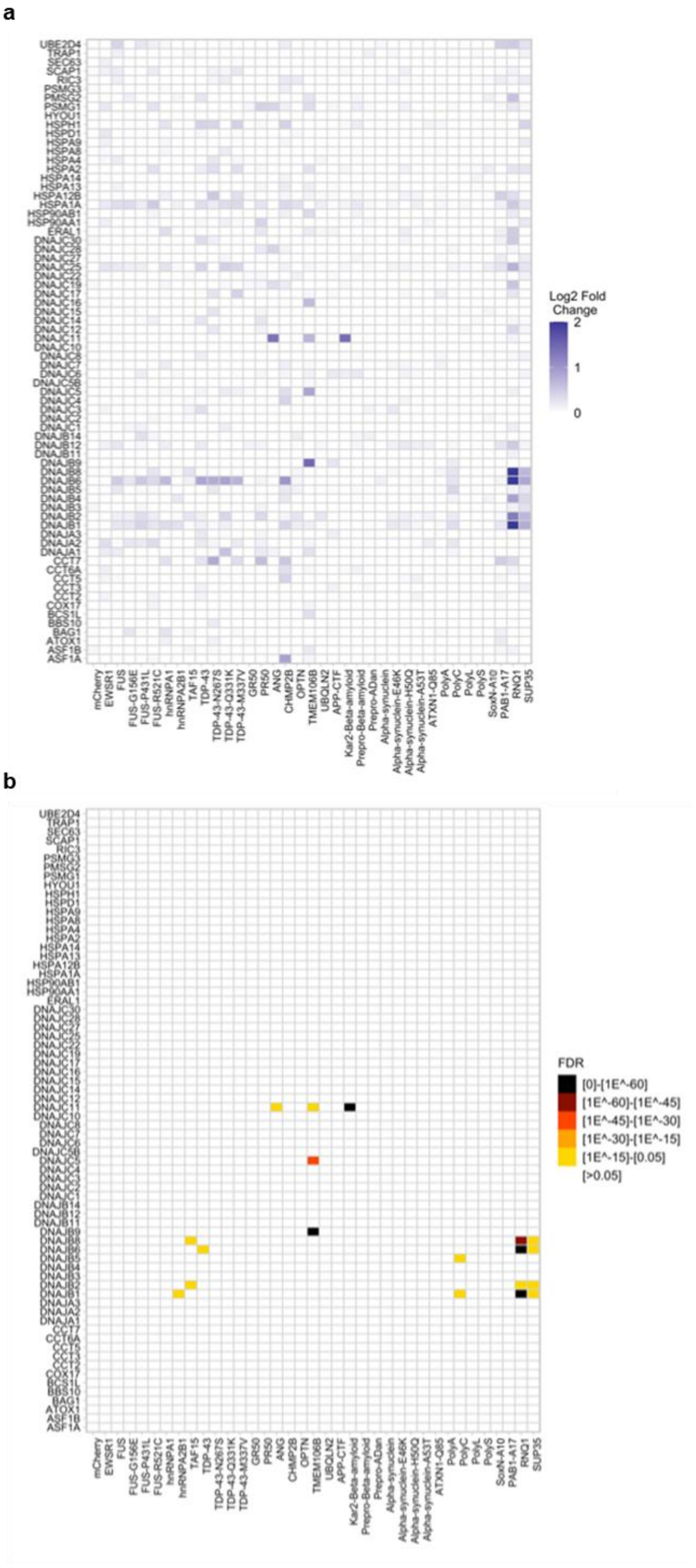

**Supplementary Figure 10. Validation of liquid culture growth assay.** The interactions tested in our pilot screen for **a.** mCherry **b.** Alpha-synuclein **c.** FUS **d.** TDP-43 **e.** RNQ1 and **f.** SUP-35 and validated with spot assays were re-tested with the liquid culture growth assay to validate its behavior. Data are shown as mean ± s.d. for three biological replicates. Comparisons were conducted with ordinary one-way ANOVA with display of comparisons of interactions with in positive changes in relative growth; ns = not significant, *P≤0.05, **P≤0.01, ***P≤0.001, ****P≤0.0001.

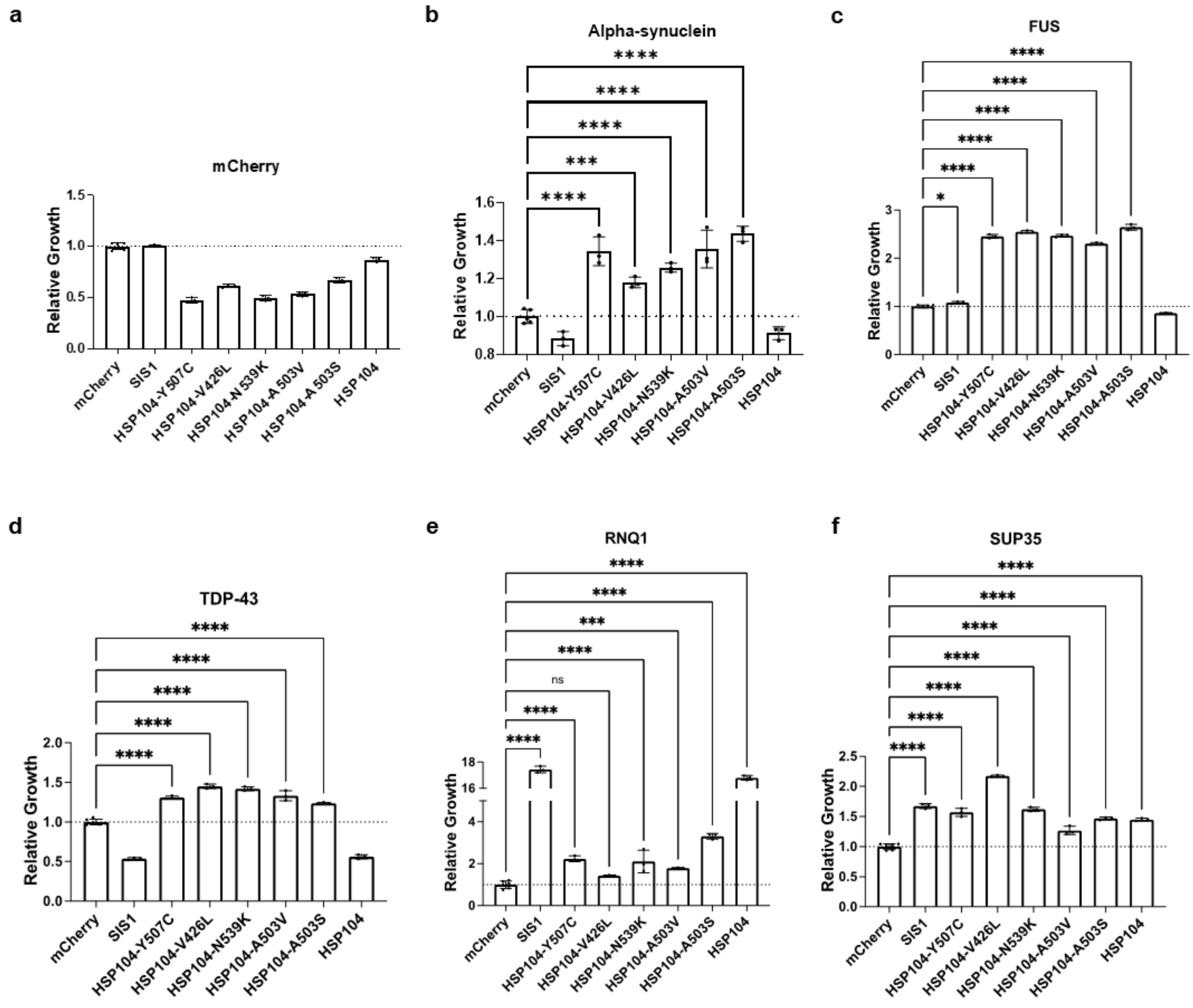

**Supplementary Figure 11. Validation of interactions not predicted to cause growth rescue.** Eleven ORFs that did not result in growth rescue in the multiplexed screen were individually tested for their ability to increase the growth rate for **a.** FUS **b.** TDP-43 **c.** EWSR1 **d.** hnRNPA1 **e.** PR50 **f.** Kar2-Abeta and **g.** Alpha-synuclein expressing yeast. Data are shown as mean ± s.d. for three biological replicates for chaperones and six biological replicates for the mCherry condition.

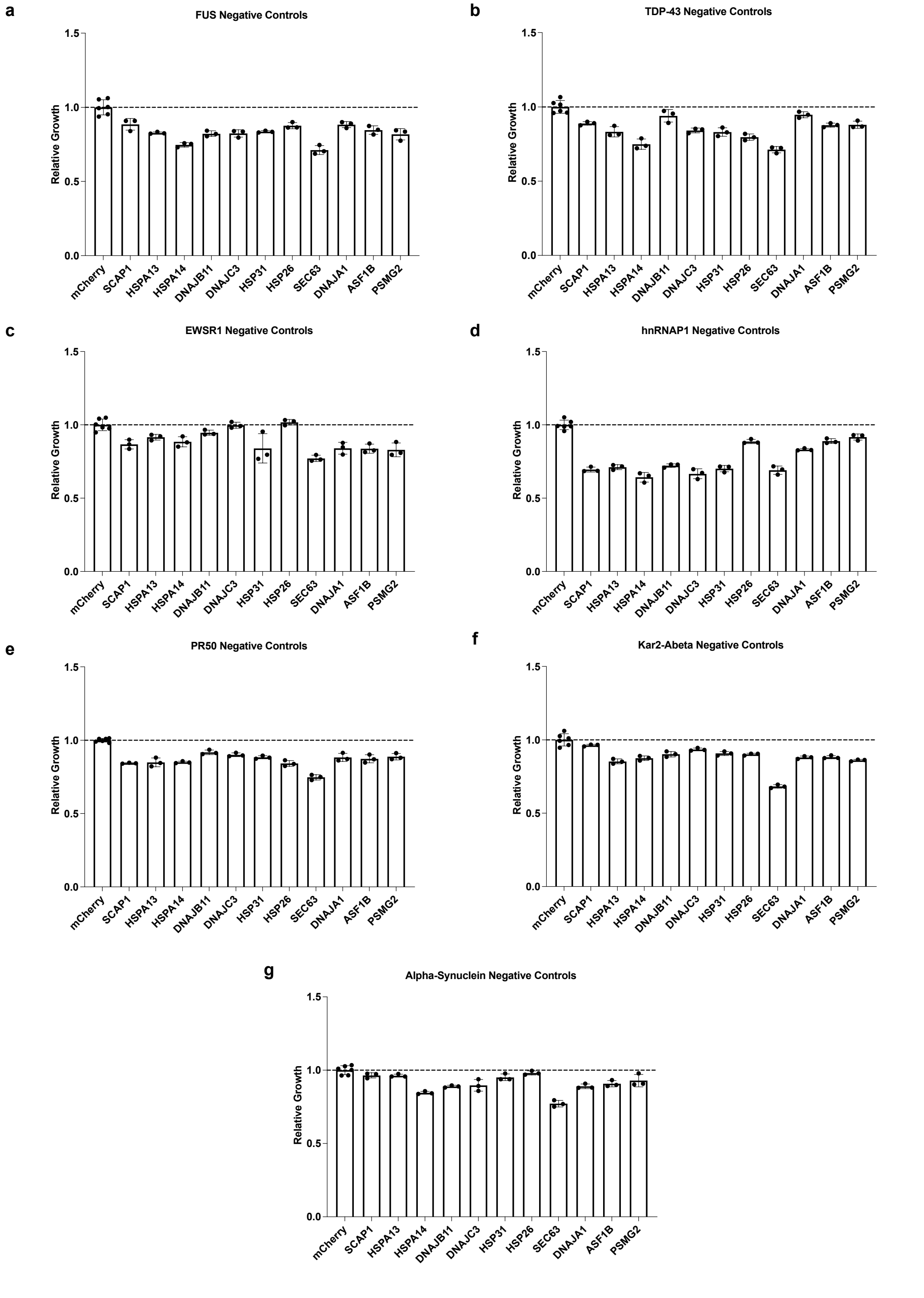

**Supplementary Figure 12. Subsampling of barcodes per model demonstrates power of redundant barcoding.** Yeast and human rescuers with called hits were reanalyzed with fewer barcoded strains included in the analysis. All hits shown in the 5-7 Barcodes/Model condition were validated.

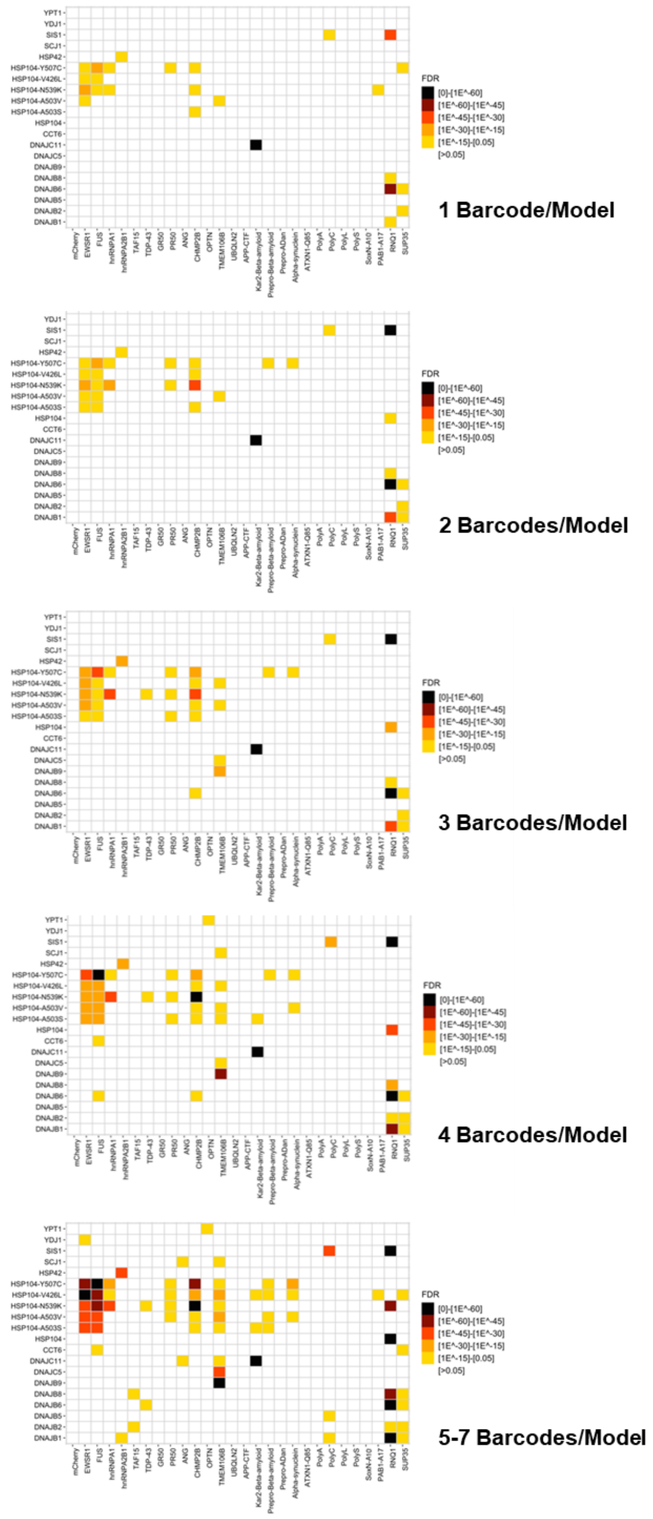

**Supplementary Figure 13. Identification and validation of interactions from the hORFeome screen.** **a.** Log2 fold change interactions between a select set of hits from the ORFome screen and the models included in the pool. Manual validation in yeast of detected interactions for **b.** ATXN1-Q85 **c.** Alpha-synuclein **d.** FUS and **e.** TMEM106B. Data are shown as mean ± s.d. for three biological replicates, independent outgrowths. Comparisons for b. and d. were conducted with ordinary one-way ANOVA and comparisons c. and d. were conducted with a two-sided Welch's t test; *P<0.05, **P<0.01, ****P<0.0001.

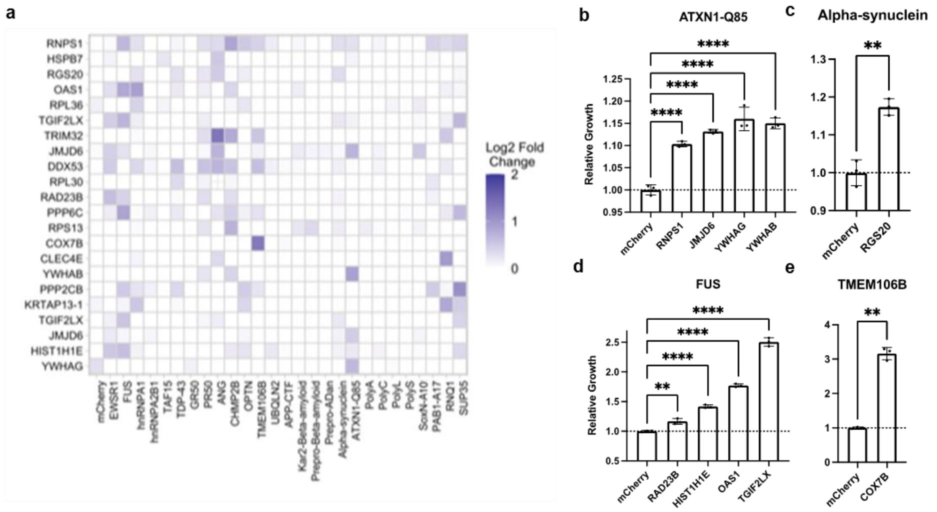

**Supplementary Figure 14. DNAJB6 shows specific activity against FUS, TDP-43, and hnRNPA1. a-c.** DNAJB6 was tested alongside other human HSP40 proteins for their ability to rescue **a.** FUS, **b.** TDP-43, and **c.** hnRNPA1 proteotoxicity in yeast. Data are shown as mean ± s.d. for three biological replicates. Comparisons were conducted with ordinary one-way ANOVA with display of comparisons of interactions with in positive changes in relative growth; *P≤0.05, ***P≤0.001, ****P≤0.0001.

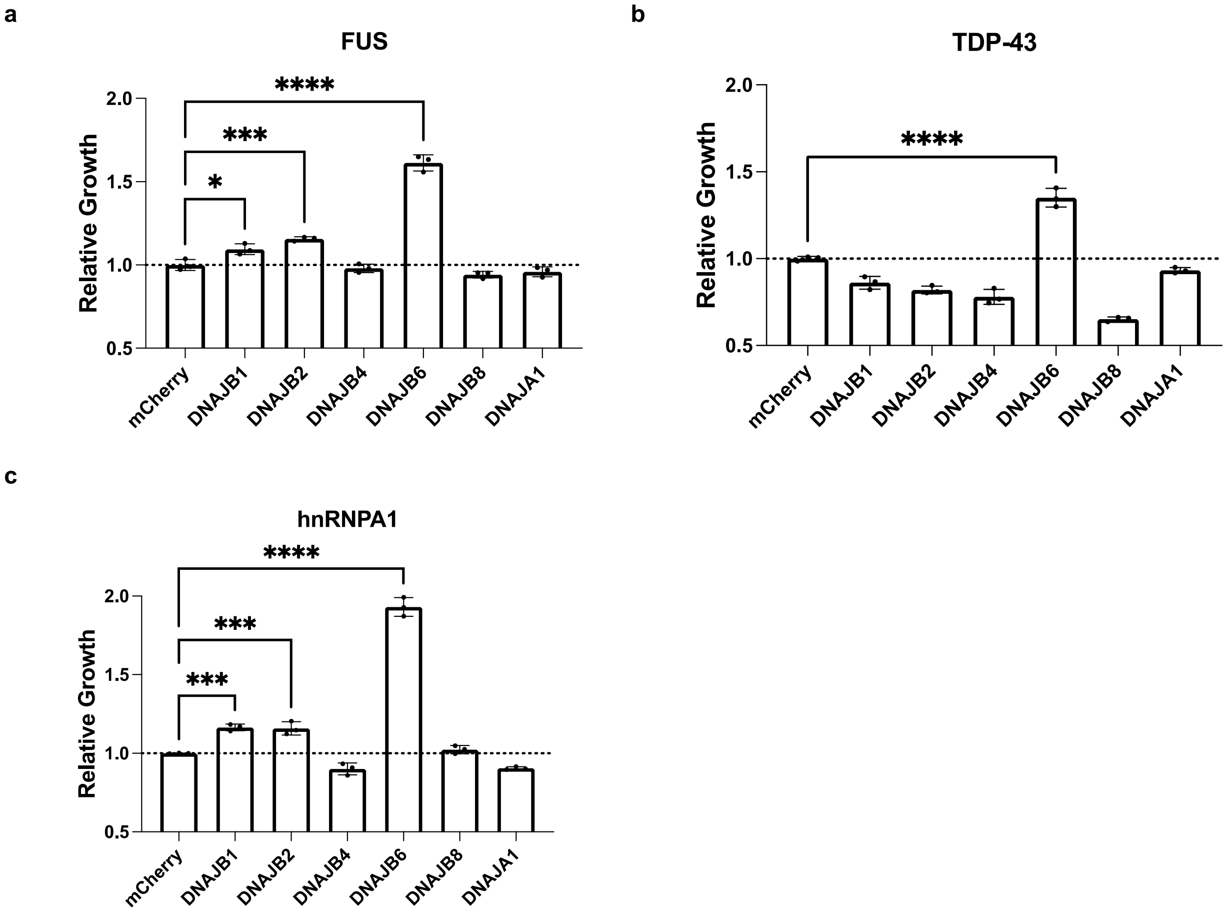

**Supplementary Figure 15. Full unprocessed blots for DNAJB6 overexpression experiments.** All individual replicate blots for FUS, TDP-43, and hnRNPA1 experiments as displayed in Figure 2b, 2c, and 2d including both antibody staining (left) and total protein Ponceau S staining (right). Red rectangles outline relevant portions of blots. All blot lanes are in the same order as presented in Figure 2b-d.

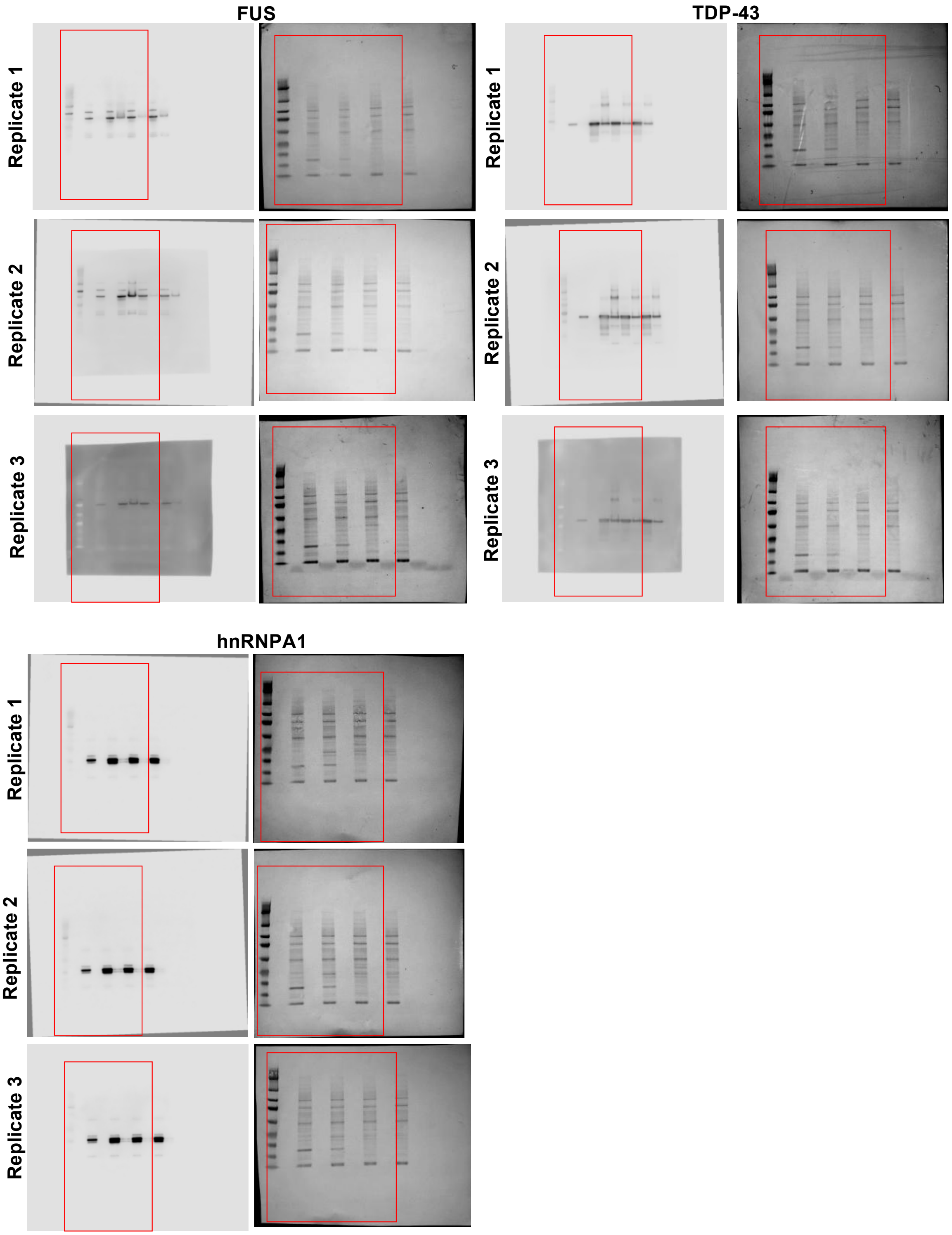

**Supplementary Figure 16. RBP overexpression selectively leads to an increase in DNAJB6 levels. a.** RNA-seq demonstrates that DNAJB6 is significantly upregulated in response to FUS or TDP-43 overexpression in HEK293T cells. Volcano plots for HEK293T expressed chaperones in cells overexpressing FUS or TDP-43 as compared to EYFP. Two biological replicates were done for each condition. **b.** Western blot and Coomassie total protein stain from HEK293T cells transfected with EYFP, FUS or TDP-43. Independent transfections were performed for each sample displayed. **c.** Quantification of DNAJB6 bands normalized to total protein detected by Coomassie staining in b. Quantification is shown for the longer DNAJB6a isoform (38 kDa) which contains a nuclear localization signal (NLS) at its C-terminus as compared to the shorter DNAJB6b isoform (25 kDa) that lacks the NLS. Comparisons for c. were conducted with ordinary one-way ANOVA; not significant = ns, **P<0.01, data are shown as mean ± s.d. for three biological replicates.

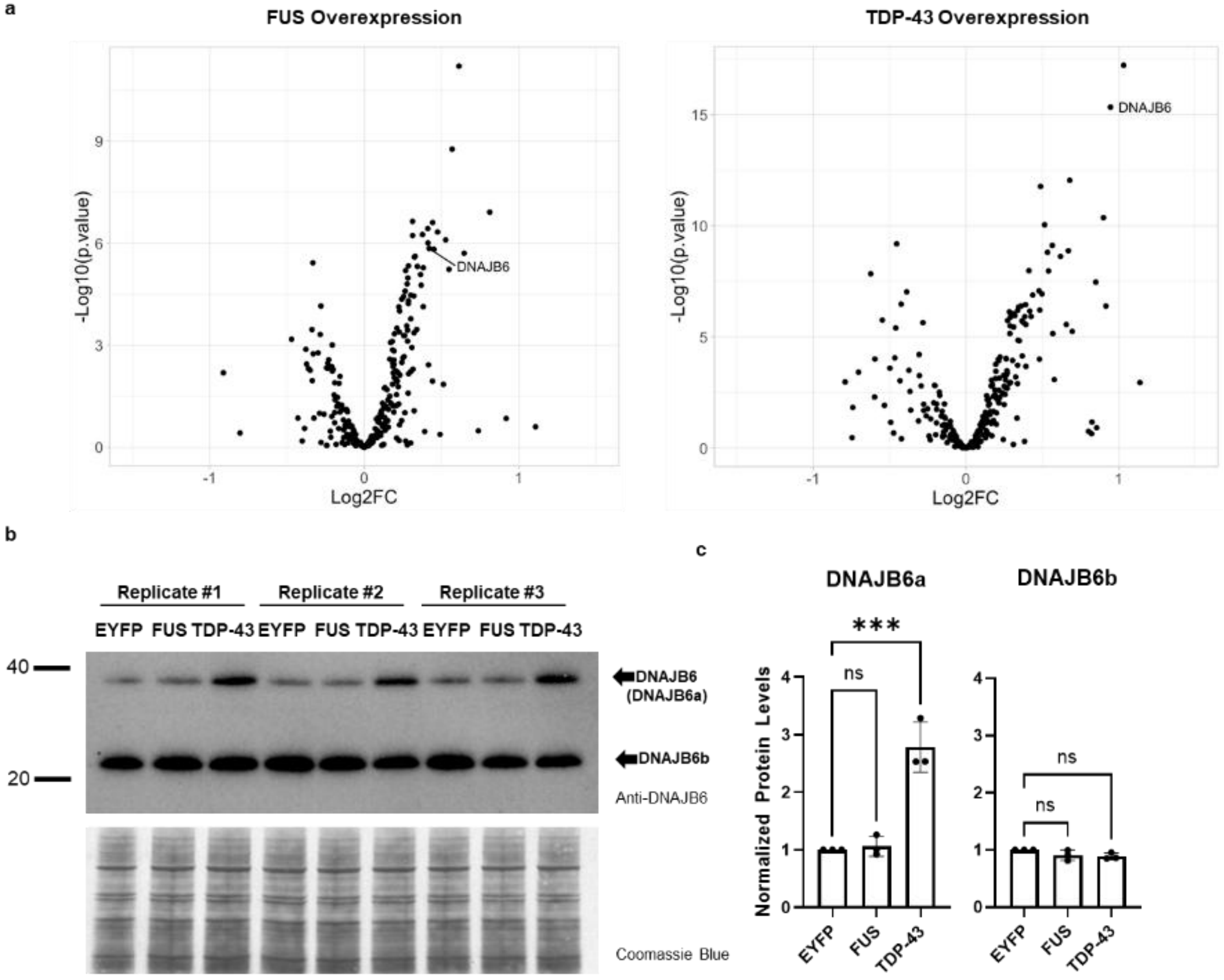

**Supplementary Figure 17. Full unprocessed blots for measuring DNAJB6 levels after FUS and TDP-43 overexpression.** All individual western blots for both DNAJB6 antibody staining (left) and corresponding Coomassie Blue staining for total protein measurement (right), as seen in Sup. Fig. 16. All blot lanes are in the same order as presented in Sup. Fig. 16 with relevant lanes outlined by a red rectangle.

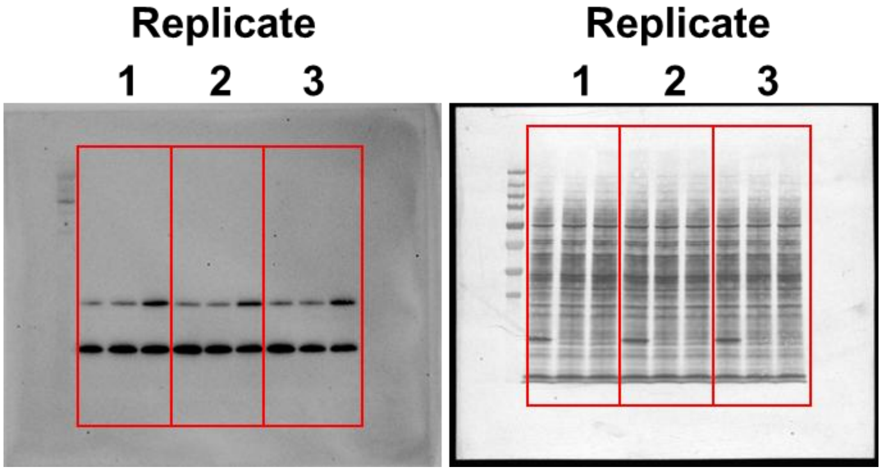

**Supplementary Figure 18. Knockout of DNAJB6 does not impact SDS solubility of endogenously expressed FUS, TDP-43, or hnRNPA1 in HEK293T cells. a.** HEK293T NTC and DNAJB6 KO cells were transfected with an EYFP expression vector and endogenous levels of TDP-43, FUS, and hnRNPA1 were assessed. **b.** Uncropped western blots for data shown in panel a with relevant lanes outlined by a red rectangle.

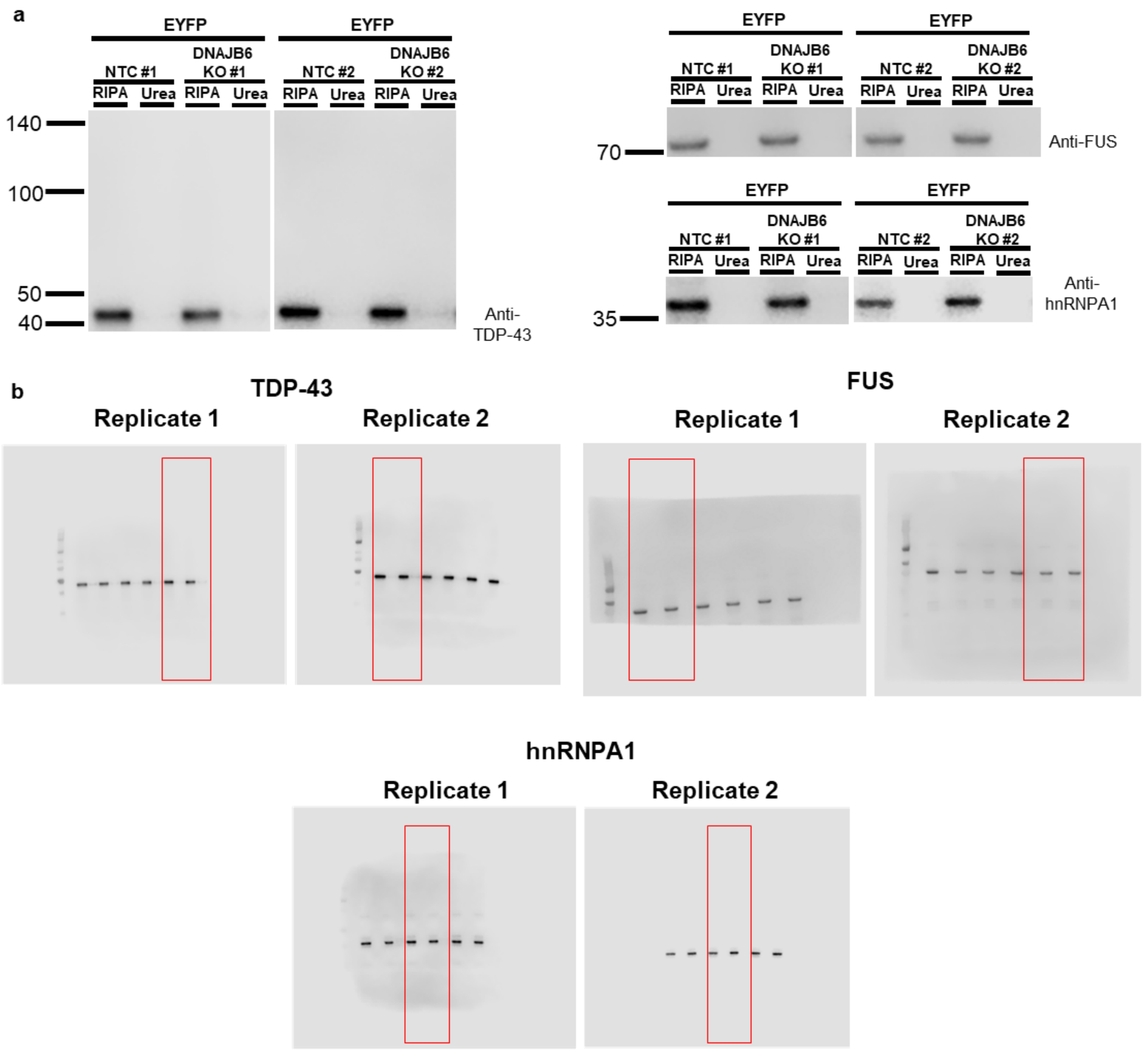

**Supplementary Figure 19. Full unprocessed blots for DNAJB6 KO experiments.** All individual replicate blots for FUS, TDP-43, and hnRNPA1 overexpression in two DNAJB6 KO lines and two corresponding NTC lines as displayed in Figure 2g, 2h, and 2i including both antibody staining (left) and corresponding Ponceau S staining for total protein measurement (right). All blot lanes are in the same order as presented in Figure 2g-I with relevant lanes outlined by a red rectangle.

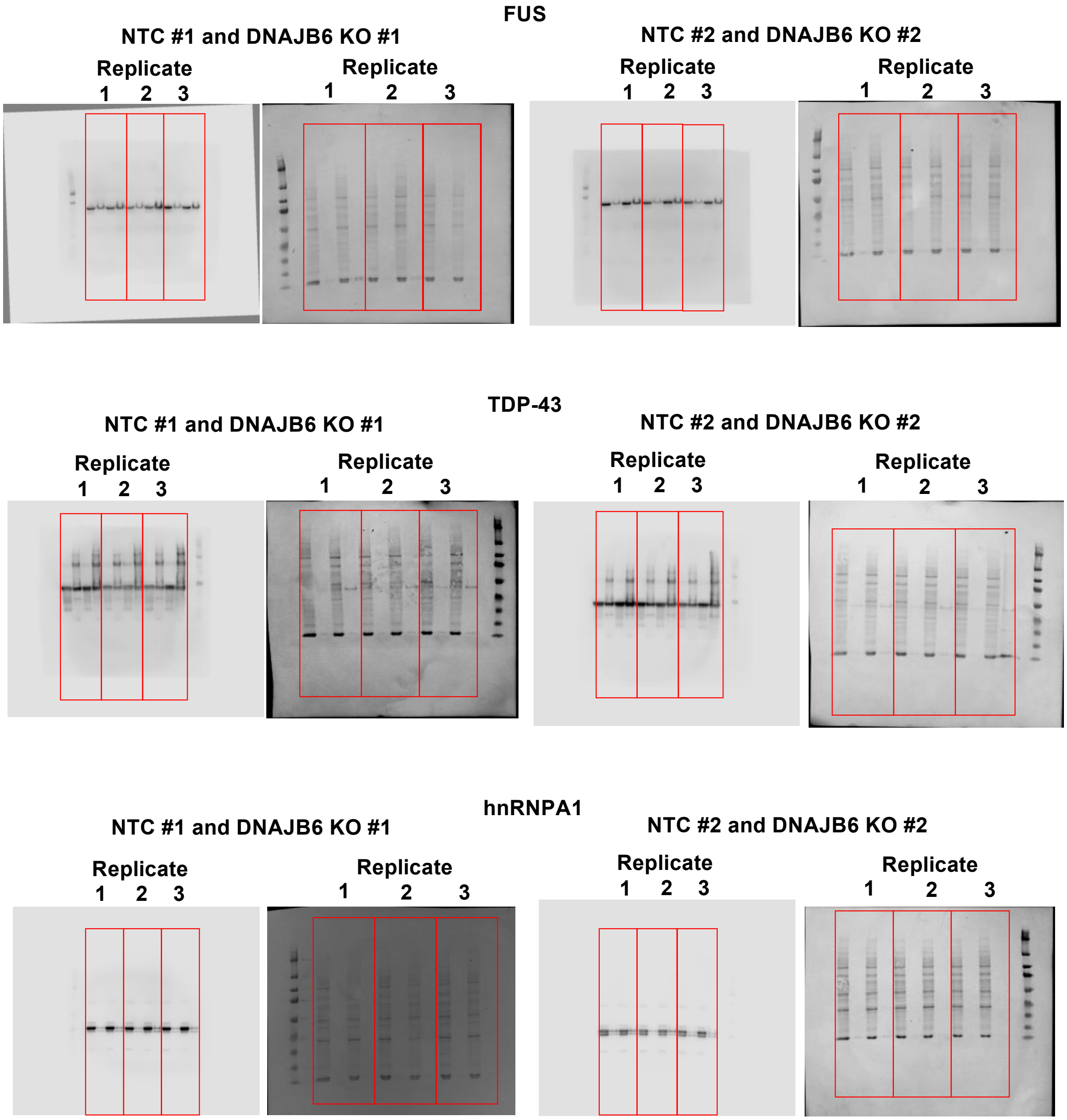

**Supplementary Figure 20. Co-immunoprecipitation (Co-IP) assays probing interactions between DNAJB6 and various RBPs. a.** Protein homogenates from HEK293T cells transfected with plasmids overexpressing untagged DNAJB6 (WT) or 3xFLAG-DNAJB6 and either FUS, TDP-43 or hnRNPA1 were used as the input for a Co-IP. **b.** Uncropped western blots for data shown in panel a.

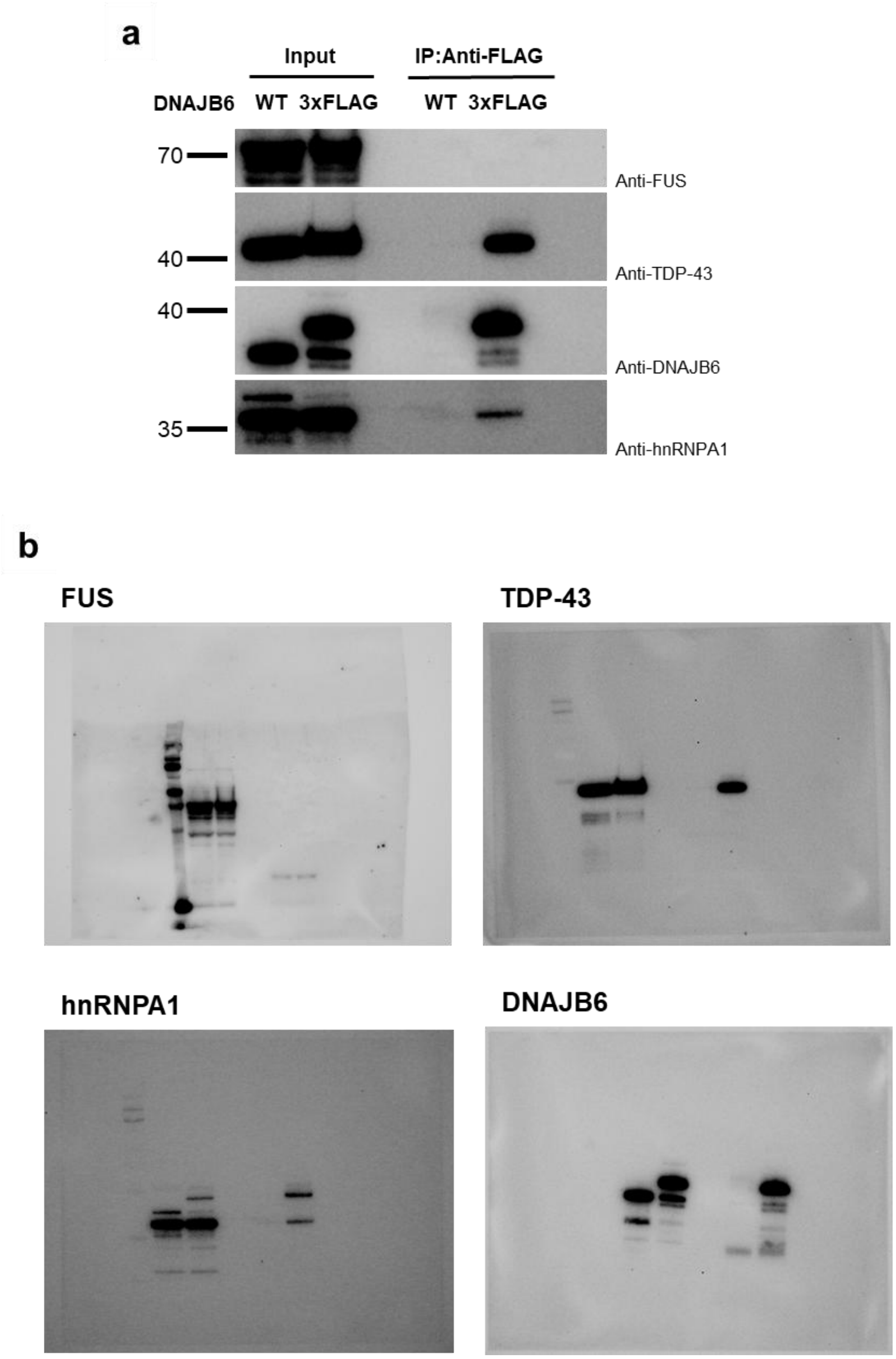

**Supplementary Figure 21. Biophysical characterization of DNAJB6 with clients. a.** DNAJB6 at 3 µM concentration undergoes LLPS at physiological salt concentrations, sample imaged 30 minutes after dilution. Scale bar = 2 µm. **b.** Ability of AF555 labeled DNAJB6 at 0.25 µM to LLPS in 500 mM NaCl and 50 mM NaCl conditions. Samples were imaged at 30 minutes. Scale bar = 5 µm. **c.** FUS-mEmerald and AF555 labeled DNAJB6 co-mingle when mixed at physiological salt concentrations and at an endogenous (6:1) ratio, 1.5 µM and 0.25 µM, respectively. Scale bar = 10 µm. Samples were imaged 20 minutes after mixing. **d.** FUS-mEmerald (1.5 µM) alone and FUS-mEmerald + DNAJB6 (1.5 µM + 0.25 µM) condensates were subjected to FRAP 30 minutes after condensate formation. **e-g.** Fluorescence lifetime measurements and statistical evaluation between mEmerald-tagged FUS proteins and mEmerald alone in the presence and absence of DNAJB6. For FUS, condensate averaged fluorescence lifetimes were measured, while for mEmerald, due to the absence of condensation, image averaged lifetimes are reported. **e-f.** Non-parametric Kruskal-Wallis test with Dunn's multiple comparison. **g** One-way ANOVA with Tukey's multiple comparison test. Error bars denote ± s.d. **h.** Illustration of custom-built confocal detection set-up employed to perform FUS dilute phase concentration measurements. The straight channel microfluidic device is mounted to a motorized stage which is connected to a 485 nm laser source and a 60x objective, where the signal is recorded using an avalanche photodiode (APD).

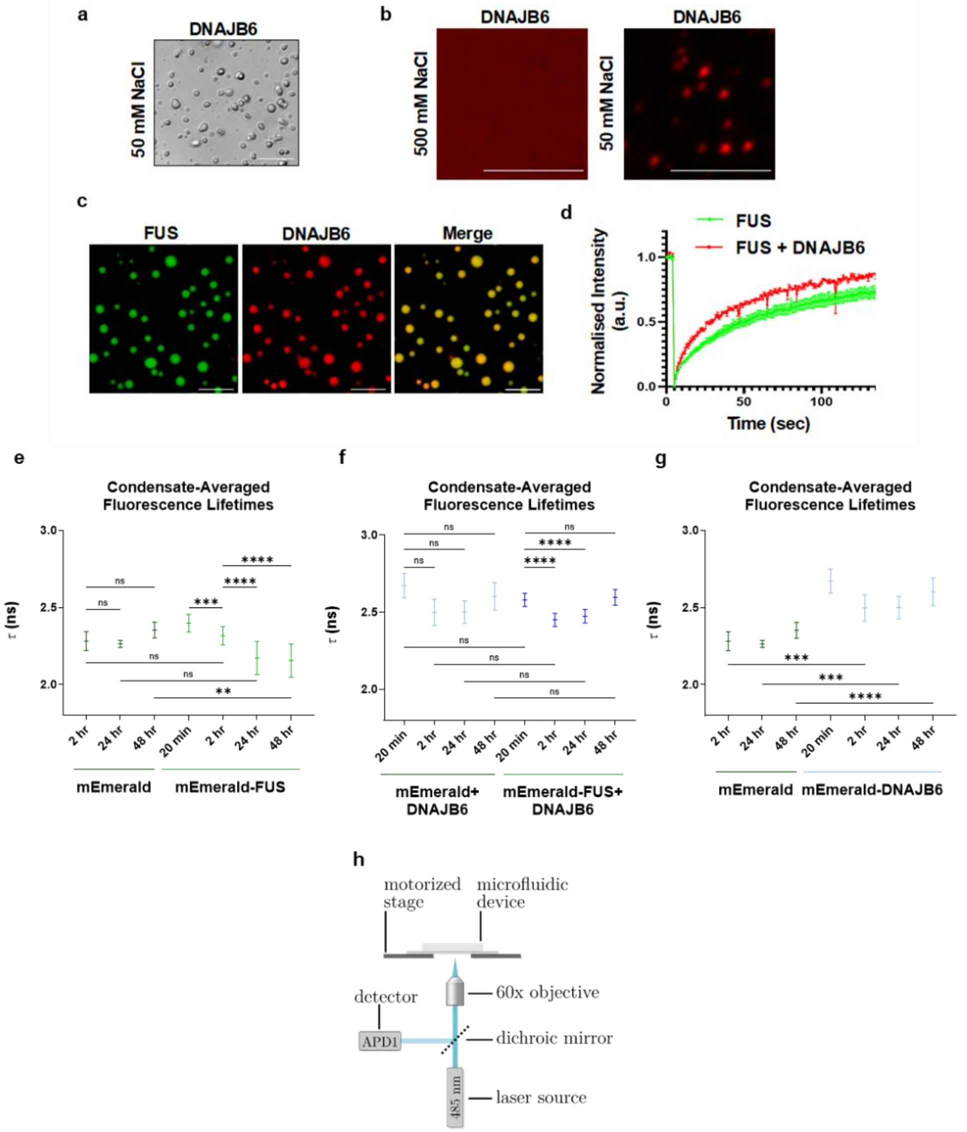

**Supplementary Figure 22: pFTAA staining quantification and AFM quantification and FTIR spectra of DNAJB6. a.** Quantification of pFTAA signal from condensates at 48 hr (each dot corresponds to the average of the signal of the condensates in a field of view, 807 condensates for mCherry-FUS and 8,976 condensates for mCherry-FUS with DNAJB6). Mann-Whitney non-parametric test p-value < 0.01. The FTIR spectra **b.** and second derivate **c.** were acquired for DNAJB6 alone to ensure the secondary structure changes detected for FUS + DNAJB6 condensates was not solely due to the presence of the additional protein component. Spectra were measured at 3 µM (~9x higher than in FUS + DNAJB6 measurements), as there was insufficient signal at lower concentrations.

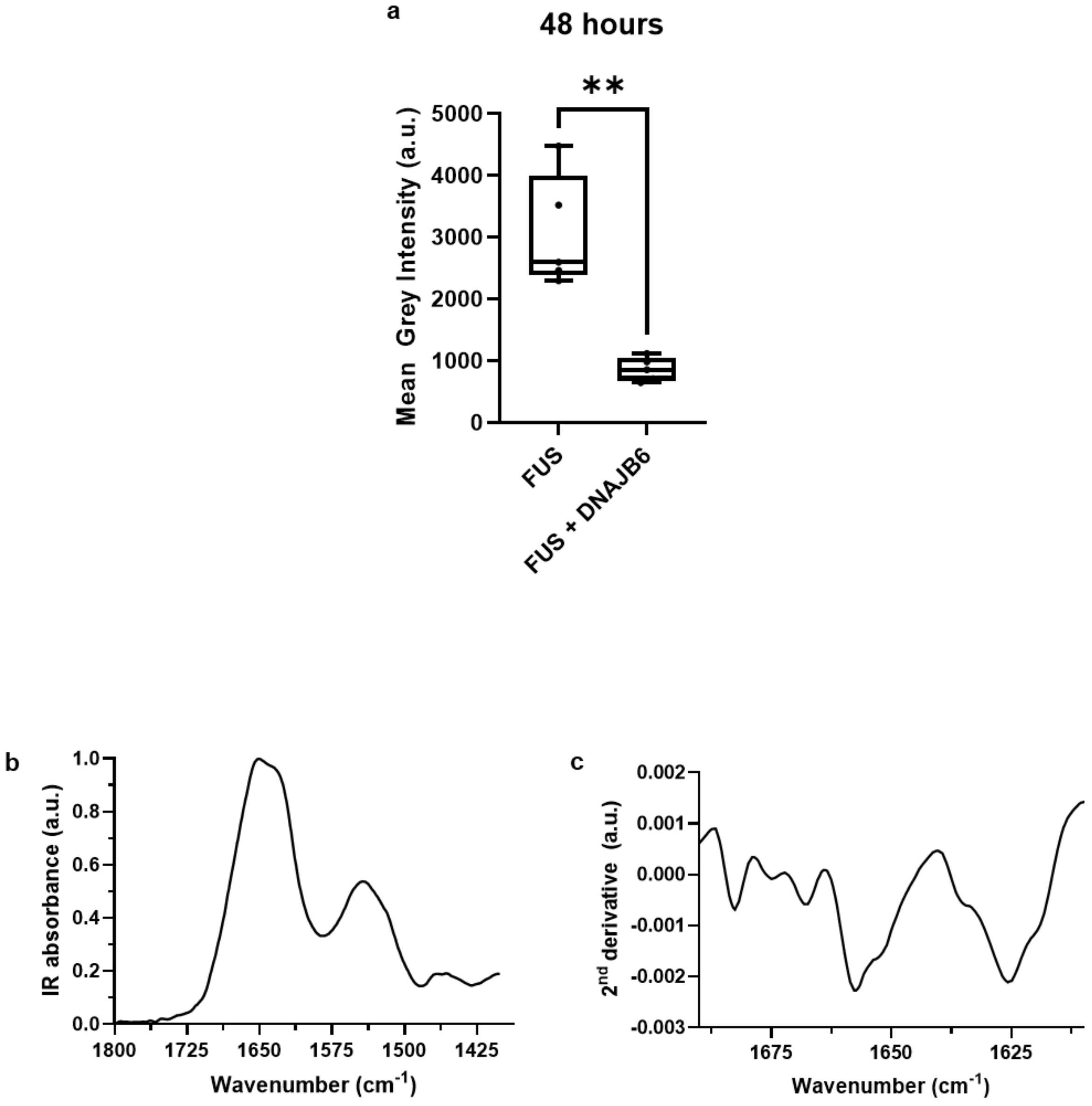

**Supplementary Figure 23. AFM-IR nano-chemical analysis on single FUS and FUS+DNAJB6 condensates.** Example for a single FUS+DNAJB6 condensate map showing **a.** 3-D morphology, **b.** IR absorption in the Amide I (1655cm-1), **c.** Contact resonance by phase locked loop (PLL) which offers a quality control on the chemical signal provided by AFM-IR. **d.** 3D morphology maps of 2 independent FUS and 2 independent FUS + DNAJB6 condensates. The fine black lines are defects in the ZnSe surface**. e.** IR spectra from 10 independent locations (each location 5 co-averaged spectra) on the condensate, **f.** their average + SE and **g.** second derivative of the amide I band to deconvolve protein secondary structure contributions. **h.** Maps of 3D morphology and **i.** second derivative of the amide I band to **j.** deconvolve fibrillar secondary structure vs gel-like condensates.

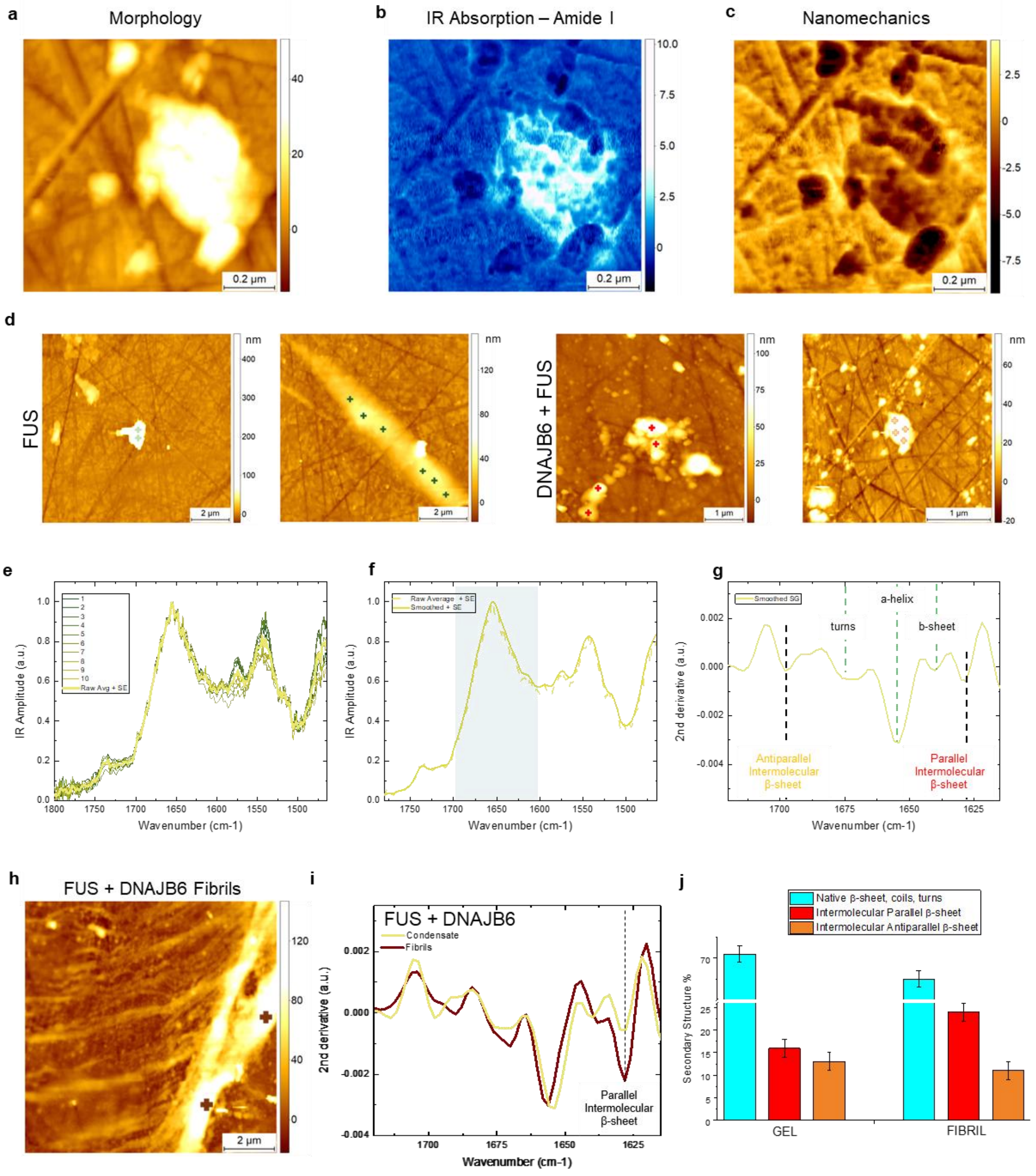

**Supplementary Figure 24. Identification of DNAJB6 domains important for activity in yeast. a-b**.Testing domain deletions and J domain H31Q loss of function point mutant for their ability to rescue the FUS expressing yeast model. ΔJ, ΔG/F, ΔS represent deletion of the J-domain, glycine-phenylalanine rich, or serine rich region of DNAJB6, respectively. Comparisons were conducted with ordinary one-way ANOVA; ****P<0.0001. **c.** Uncropped western blots for data shown in panel b.

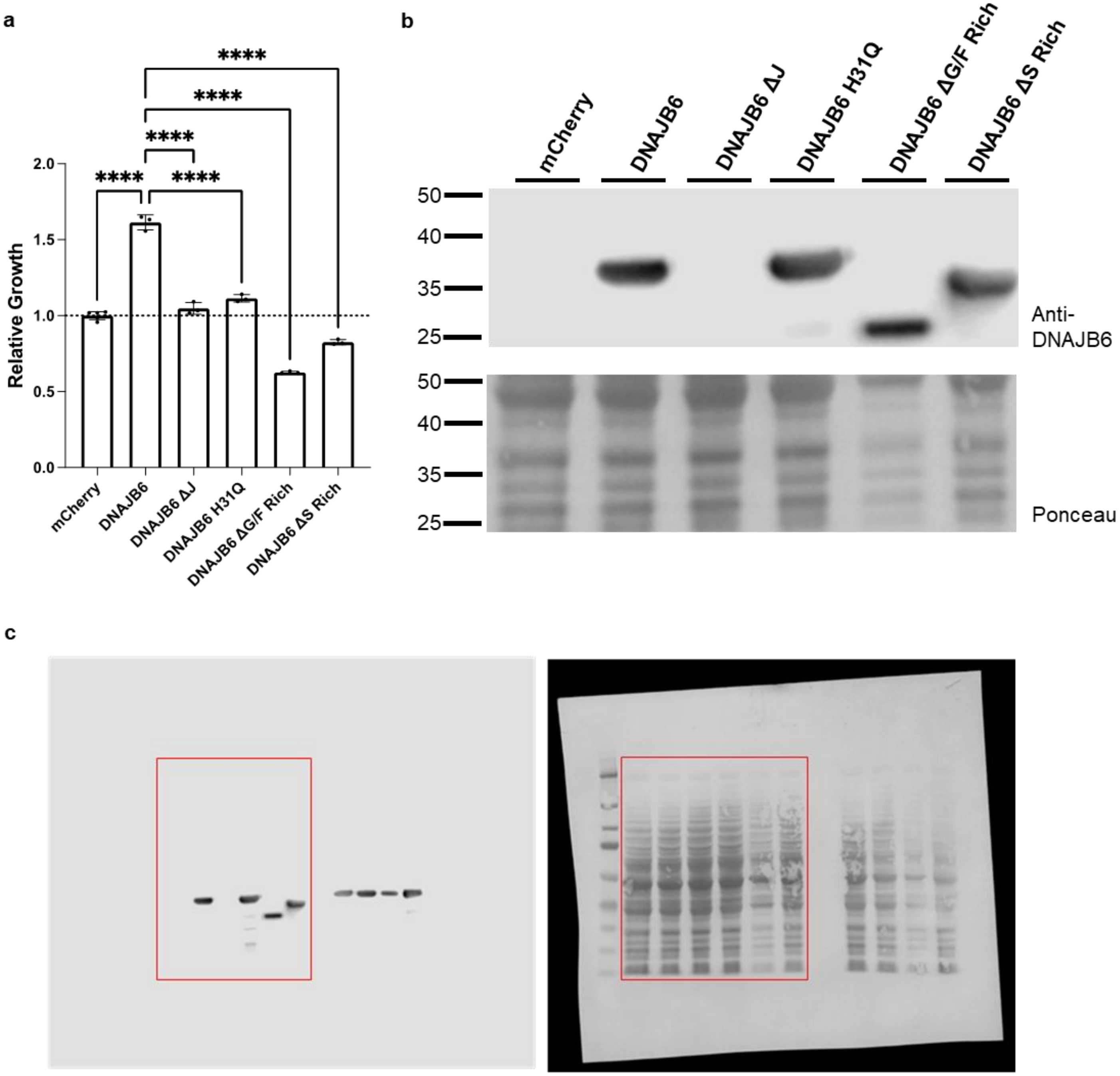

**Supplementary Figure 25. DNAJB6-HSP70 interaction is required for RBP solubilization in cells.** Representative images comparing SDS-soluble (RIPA) and SDS-insoluble/Urea-soluble fractions extracted from cells co-expressing WT DNAJB6 or DNAJB6 H31Q with **a.** FUS **b.** TDP-43 **c.** hnRNPA1. **d.** Quantification of the urea soluble species normalized to total protein detected by Coomassie staining in a-c. All statistical tests were conducted with two-sided Welch's t test; *P≤0.05. Data are shown as the mean ± s.d. for the three separate transfections and extractions.

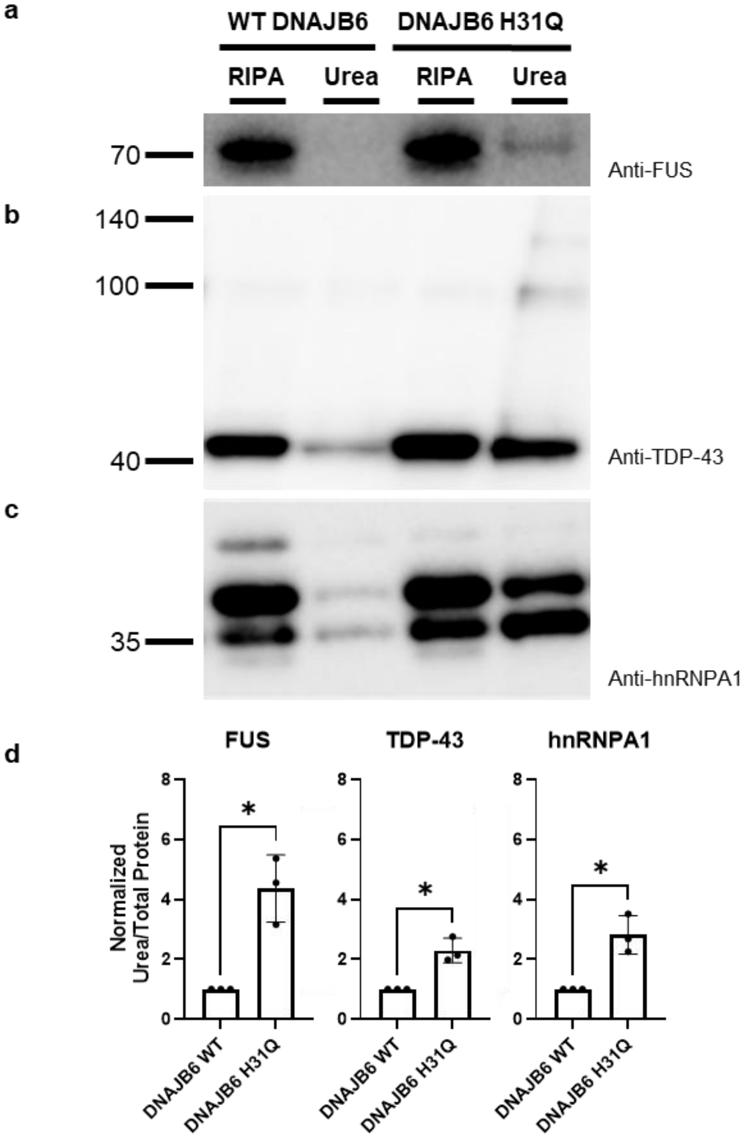

**Supplementary Figure 26. Full unprocessed blots for dependence of DNAJB6 on HSP70 for rescue in HEK293T cells.** All individual replicate blots for FUS, TDP-43, and hnRNPA1 overexpression in HEK293T cells as displayed in Sup. Fig. 25 including both antibody staining (left) and corresponding Coomassie Blue staining for total protein measurement (right). All blot lanes are in the same order as presented in Sup. Fig. 25 with relevant lanes outlined by a red rectangle.

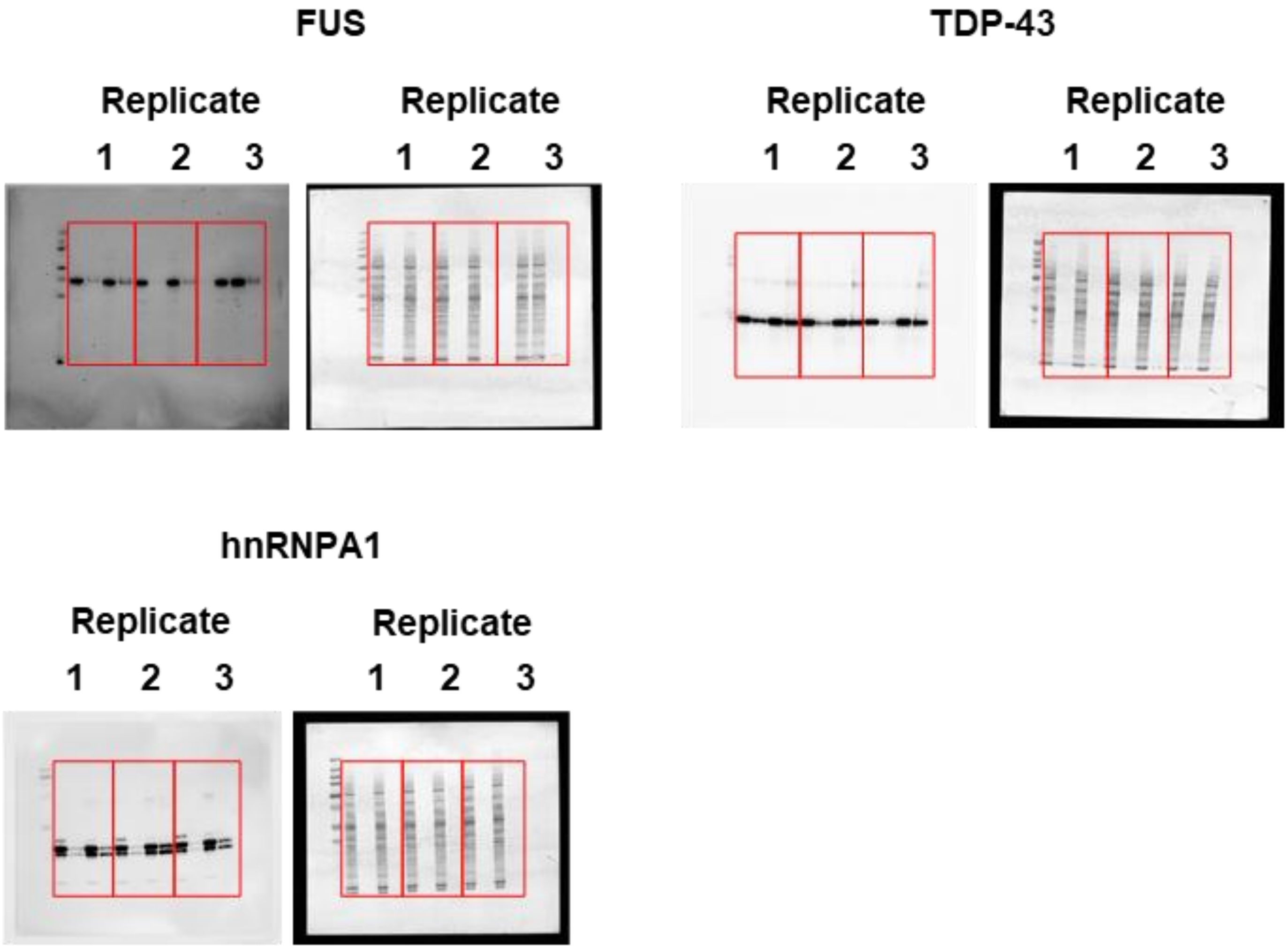

**Supplementary Figure 27. Correlation statistics between biological replicates of deep mutational scan of DNAJB6. a.** Correlation between log2 fold changes for all amino acid changes at all positions tested in the deep mutational scanning approach. **b.** Correlation between log2 fold changes for amino acids in each set of 14 amino acids analyzed together in one sequencing batch.

a

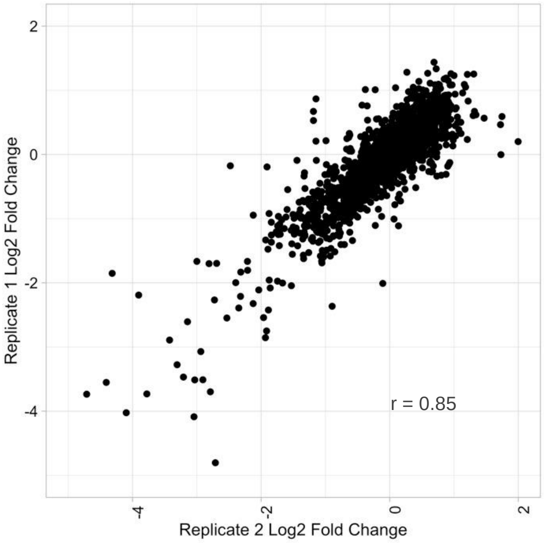


b

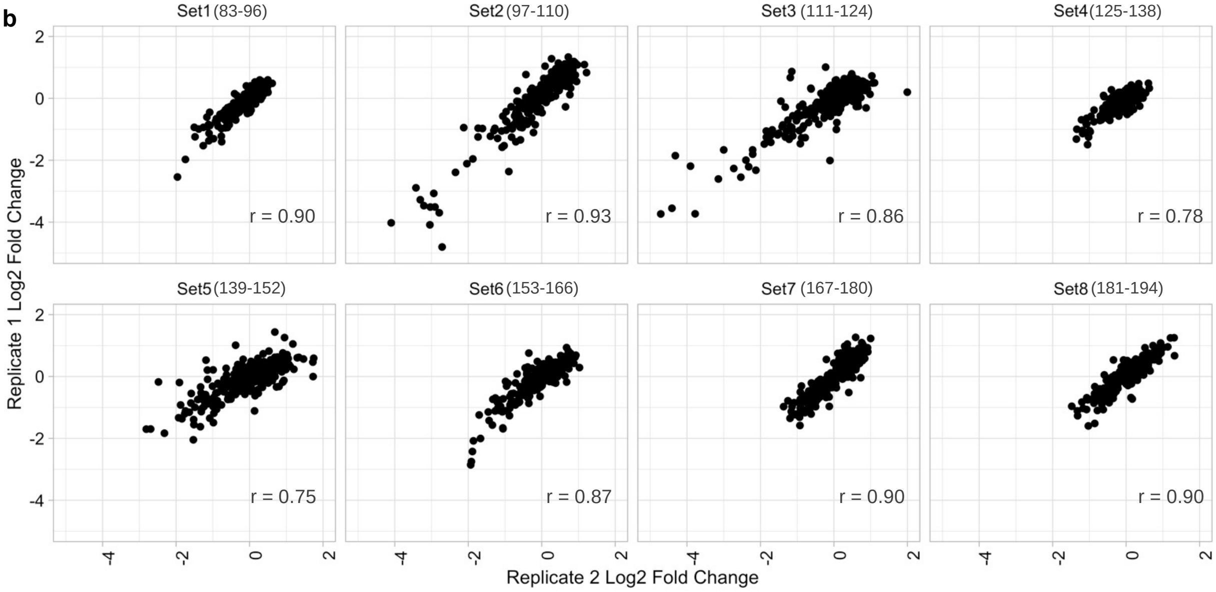

**Supplementary Figure 28. Mutations in DNAJB6 can affect its intrinsic toxicity and ability to rescue RBP toxicity.** Individual growth assays for several DNAJB6 variants expressed alone in yeast cells are shown. FUS and EYFP, a toxic and non-toxic protein, respectively were tested at the same time to provide a comparator. Many but not all DNAJB6 variants that show a loss of FUS and TDP-43-mediated rescue also show enhanced toxicity on their own as compared to WT DNAJB6. Comparisons were conducted with ordinary one-way ANOVA; ns not significant, ***P<0.001,****P<0.0001

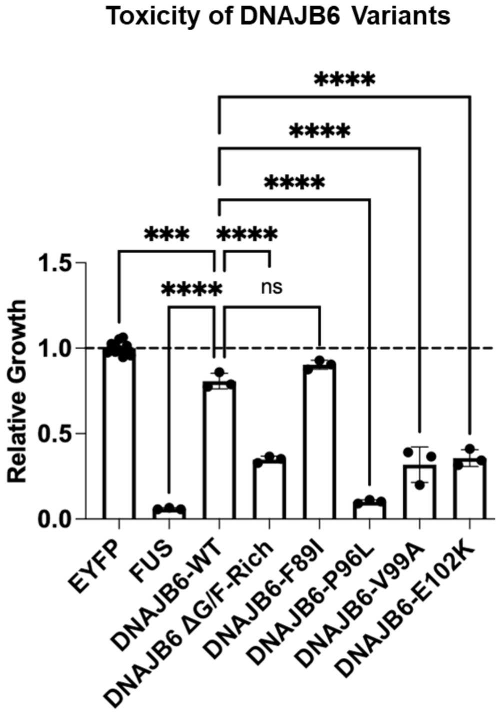

**Supplementary Figure 29. Increasing amount of FUS plasmid transfected increases the amount of SDS-insoluble, Urea-soluble species. a.** Different doses of FUS expression plasmid were transfected and protein was harvested 48 h after transfection to assess the solubility of FUS. **b.** Uncropped western blots for data shown in panel a with the relevant lanes outlined by a red rectangle.

a

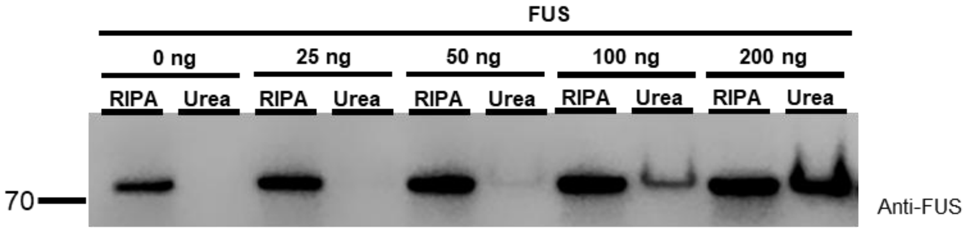


b

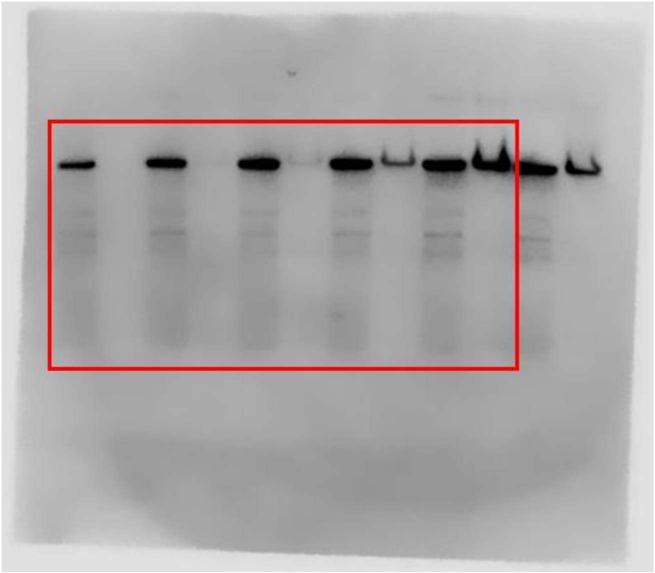

**Supplementary Figure 30. Full unprocessed blots for hyperactive DNAJB6 experiment.** Uncropped Western blot for Fig 7d is shown below, lanes are in the same order as in the main figure.

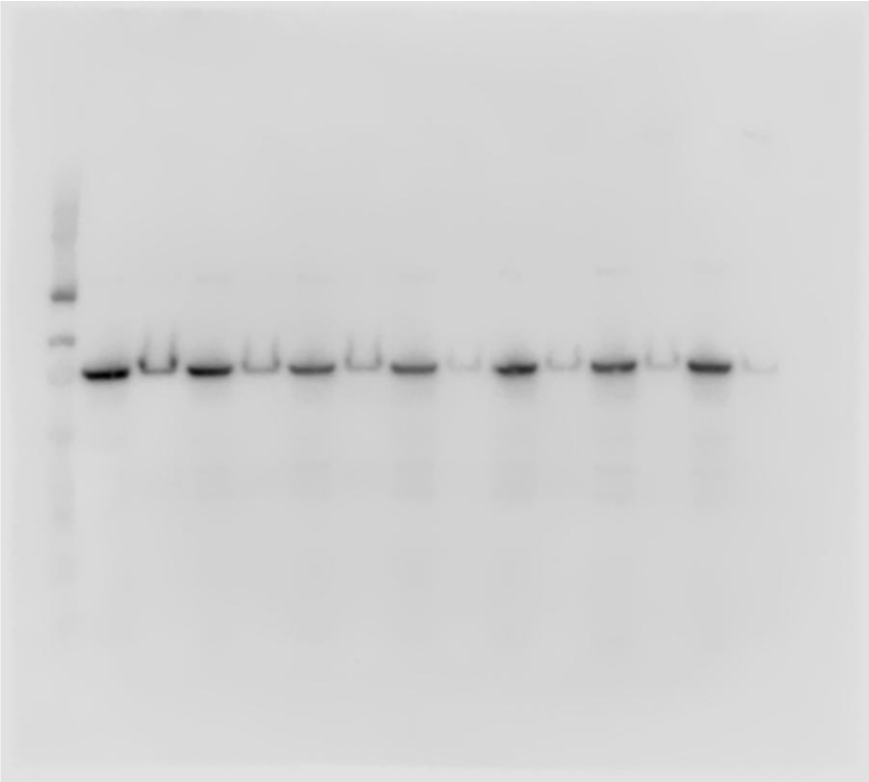

**Supplementary Figure 31. Validation of DNAJB6 expression upon exposure to ChAT-Cre. a.** AAV9-CAG-Lox-Stop-Lox-3xFLAG-DNAJB6 constructs robustly express 3xFLAG-DNAJB6 in motor neurons upon exposure to Cre driven by the ChAT promoter. **b.** Overlay of DNAJB6 and ChAT staining upon activation of DNAJB6 expression by ChAT-Cre. Scale bars represent 50 microns.

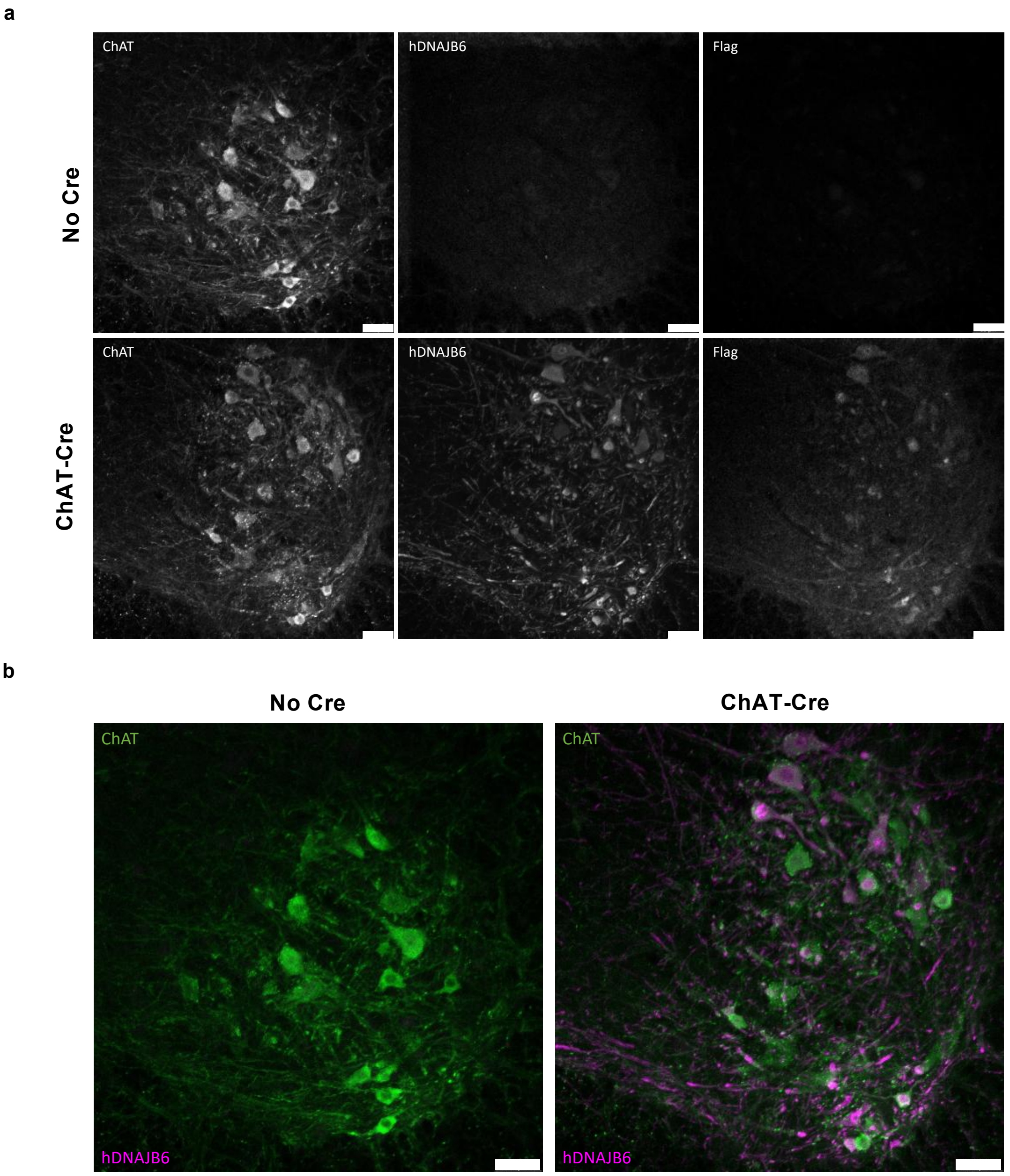

**Supplementary Note 1**

To begin to establish the feasibility of multiplex high-throughput screening, we first needed to determine the reproducibility of all the required steps within the pipeline. Towards this goal, we assembled a pilot pool composed of 117 DNA-barcoded yeast strains. Among the barcoded strains in the pool were several proteotoxic models with known genetic rescuers such as yeast prions RNQ1 and SUP35, and NDD models such as FUS, TDP-43, and alpha-synuclein. Also included in this pilot pool were other proteotoxic models selected to represent a range of different strengths of toxicity to assess how variation in the amount of growth arrest caused by a model (i.e. mild, moderate, and strong), affects the reproducibility of our system. Taking advantage of the scalability of the DNA-barcoding and to help control for variation at the biological and technical levels, each model was transformed into 3 unique isogenic DNA-barcoded strains (i.e. redundantly barcoded). This allows each barcoded variant of the same model to serve as an internal biological replicate, and for the collective behavior of all barcodes associated with the same model to be used to determine the effects of each tested genetic modifier.

The first set of experiments that were performed was testing whether *en masse* mating and selection of the pilot DNA-barcoded pool was consistent when performed across multiple wells each mated to the same control rescuer strain. We observed strong correlation between separately mated pools, suggesting relative barcode abundance is preserved through mating and selection (Sup. Fig. 2a-b). We next determined whether individually mated and selected diploid pools resulted in a reproducible behavior for all members of the library when inoculated into inducing media and allowed to grow back to saturation. We observed strong correlation between separately mated, selected, and outgrown pools. These data suggest that the pool shows a consistent behavior across replicate experiments and that endpoint measurements of barcode abundance can be used to make comparisons between control rescuer and active rescuer wells (Sup. Fig. 2c-d).

**Supplementary Note 2**

Using the pilot pool, we tested whether altering the relative abundance of particular strains in the pool might improve our ability to detect known, literature-reported interactions between molecular chaperones and the library of proteotoxic models[1,2,3]. The "All" pooling strategy evenly mixed all 117 strains. The "Low" pooling strategy evenly mixed all strains but excluded a number of control yeast strains expressing proteins that lack toxicity (e.g. enhanced yellow fluorescent protein) to enable more division opportunities before the pool reached growth saturation. The "Skew" pooling strategy mixed all 117 strains but seeded strong and moderately toxic models at a higher initial abundance compared to the mild and non-toxic models. This was done by first performing a spot assay to determine the relative growth rate of each strain in the pool. Then, strains were pooled at either a 15:1, 10:1, 5:1, or 1:1 ratio depending on the degree of growth suppression, with the most toxic strains given the highest initial abundance. We observed comparable performance between the Low and All pools, with 16/17 and 14/17 literature-reported positive controls demonstrating positive log2 fold change when the behavior of all the DNA-barcodes associated with the same model were averaged (Sup. Fig. 3a). In sharp contrast, the Skew pool showed the worse performance detecting only 12/17 positive control interactions, along with showing overall lower log2 fold changes as compared to the Low and All pools.

Upon further examination of the resulting data, a stronger correlation between biological replicates using the Low pooling strategy mated to the same benign rescuer over other strategies was observed (Sup. Fig. 3b). Additional analysis was performed in which the relationship between the coefficient of variation (CV) of a barcode and its mean relative abundance in the pool was examined. As previously shown in both RNA-sequencing and microbiome sequencing datasets, low abundance members in a mixed pool tend to show higher variance in their abundance values, which we hypothesize may render more toxic models within the pool (which are rapidly depleted during outgrowth) more variable[4,5]. The ability of a pooling strategy to reduce variability at all sampling levels, in particular those with lower abundance, suggests it should have improved performance and increased sensitivity to detect real interactions. The Low pooling strategy was generally associated with lower variability for barcodes at all relative abundances. The Skew strategy did reduce the variability of lowly abundant barcodes primarily associated with highly toxic models compared to the All pooling strategy, but was also associated with higher variability for less toxic, generally more abundant models possibly as a result of their lower initial seeding (Sup. Fig. 3c).

Taking the Low pooling strategy forward, we assessed sources of biological and technical noise. For this study, we considered a biological replicate to require a separate mating, selection, outgrowth, DNA

harvest, and PCR amplification for sequencing. We considered technical replicates to be separate PCR amplification reactions performed on the same harvested DNA for sequencing. We observed relatively minor sources of both biological and technical variation (Sup. Fig. 4a). We also assessed whether averaging between multiple biological or technical replicates improved the reproducibility of the screen by reducing the CV ~ relative abundance relationship of barcoded strains. Averaging relative abundances of barcode strains between multiple replicates reduced the variability of barcoded strains, with averaging between 2 replicates conferring a similar advantage to averaging between 3 replicates (Sup. Fig. 4b-c). This suggested that a screening paradigm that adopts the Low pooling strategy with two biological replicates for each well and two technical replicates for each biological replicate is optimized for sensitive detection of genetic modifiers of proteotoxicity. We tested whether the Low pooling strategy along with two biological or technical replicates improved performance over the initial pilot experiment and observed that log2 fold changes were stronger and captured all known interactions (Sup. Fig. 5a). We validated, via spot assay, all potential interactions within this pilot interaction space and observed strong concordance with screen data (Sup. Fig. 5b-g)

**Supplementary Note 3**

The initial variance modeling data suggested that lower abundance members of the pool are highly variable and would restrict the assay to detecting only strong interactions for these models. We hypothesized that merging information between isogenic redundantly barcoded strains would help improve the detection of mild and moderate interactions for lower abundance pool members. Using the prior association of CV ~ relative abundance, we modeled the necessary fold change in order to detect statistically significant enrichment of models while also accounting for the large degree of multiple hypothesis testing when implementing the approach. To model the required fold change to detect significant interactions at an $\alpha$ = 0.05 with a pool of 50 models, we determined the multiple hypothesis corrected Z-score necessary to reach significance with a Bonferroni correction. From this Z-score, we derived the necessary fold change required to reach significance from the CV at each relative abundance. To model sharing information between barcodes, we used Stouffer's Z-score method to simulate the required individual Z-scores necessary for significance when these Z-scores are combined. Without information sharing between isogenic redundantly barcoded strains containing the same model, greater than 2-fold change in abundance was necessary for significance for lowly abundant barcodes. Our modeling suggested that information sharing between isogenic strains representing the same model would enable more sensitive detection of weaker interactions, similar to how information is shared between multiple gRNAs in CRISPR screens to identify essential genes[6]. By pooling information between 5 or more isogenic strains, we determined that rescuers that increased the abundance of a model within the mixed pool by 1.5 fold could be detected with statistical significance (Sup. Fig. 6). With this approach, we observed that the benefits of redundant barcoding scaled faster than the penalties of multiple testing, suggesting that additional redundant barcoding is favorable for sensitive detection of interactions.

For each model, we assembled 5-7 individual barcoded strains and validated equal growth between isogenic strains containing the same model but different barcodes (Supplementary Data 1). The final pool is comprised of 45 total models. Twenty-three models represent human NDD-associated proteins. An additional 9 are clinical variants of disease-associated proteins that lead to an increase in the incidence of disease. The remaining models represent poly-alanine repeat expansion models (4), yeast prions (2), Non-NDD-associated proteins that cause growth defects when expressed and serve as assay controls (5), and finally non-toxic controls (2). We pooled a total of 302 barcoded strains and assessed the CV ~ relative abundance relationship with this new pool to determine the proper read depth. We observed a similar CV ~ relative abundance relationship between this larger pool and the pilot pool used to optimize our approach. We hypothesized that increasing the read depth of each well may also reduce the CV of lowly sampled barcodes. However, we observed similar CV ~ relative abundance profiles, with 128,000

or greater reads per well demonstrating the minimum read depth required to gain most of the benefits of increased read depth in terms of CV ~ relative abundance and number of lowly sampled barcodes that are captured (Sup. Fig. 7a-b). This suggests that lowly abundant barcoded strains may retain inherent variance as a result of the degree of proteotoxicity and growth suppression they experience. At this level of sequencing depth, 24 96-well plates can be sequenced on a single Illumina NextSeq 75bp High Output run, with an approximate cost of $0.70 per screened well.

## References


1. Jackrel ME, DeSantis ME, Martinez BA, et al. Potentiated Hsp104 variants antagonize diverse proteotoxic misfolding events. *Cell*. 2014;156(1-2):170-182. doi:10.1016/j.cell.2013.11.047

2. Sondheimer N, Lopez N, Craig EA, Lindquist S. The role of Sis1 in the maintenance of the [RNQ+] prion. *EMBO J*. 2001;20(10):2435-2442. doi:10.1093/emboj/20.10.2435

3. Bagriantsev SN, Gracheva EO, Richmond JE, Liebman SW. Variant-specific [PSI+] infection is transmitted by Sup35 polymers within [PSI+] aggregates with heterogeneous protein composition. *Mol Biol Cell*. 2008;19(6):2433-2443. doi:10.1091/mbc.e08-01-0078

4. Barlow JT, Bogatyrev SR, Ismagilov RF. A quantitative sequencing framework for absolute abundance measurements of mucosal and lumenal microbial communities. *Nat Commun*. 2020;11(1):2590. doi:10.1038/s41467-020-16224-6

5. McIntyre LM, Lopiano KK, Morse AM, et al. RNA-seq: technical variability and sampling. *BMC Genomics*. 2011;12:293. doi:10.1186/1471-2164-12-293

6. Li W, Xu H, Xiao T, et al. MAGeCK enables robust identification of essential genes from genome-scale CRISPR/Cas9 knockout screens. *Genome Biol*. 2014;15(12):554. doi:10.1186/s13059-014-0554-4
