# Supplementary material for: Multiplex neurodegeneration proteotoxicity platform reveals DNAJB6 promotes non-toxic FUS condensate gelation and inhibits neurotoxicity
Source: Nat Commun. 2025 Nov 21;16:10285. doi: 10.1038/s41467-025-65178-0 (PMC12638947; doi:10.1038/s41467-025-65178-0)
Supplement: Supplementary file 2 — Description of Additional Supplementary Files [file 41467_2025_65178_MOESM2_ESM.pdf]

## Description of Additional Supplementary Files

**Supplementary Data 1. Models included in pool.** Names, sequences, and 20 bp barcodes of models in the pool. Growth and passaging conditions for each model's secondary validation are also included.

**Supplementary Data 2. Results of chaperone screen.** Primary data from analysis pipeline for each interaction in the chaperone screen data set. Log2FC = Log2 fold change, FDR = False Discovery Rate.

**Supplementary Data 3. Raw count data for chaperone screen.** Count matrix for all barcoded yeast strains across each well tested for the chaperone screen.

**Supplementary Data 4. Results of secondary validation of called hits and suspected interactions from the chaperone screen.** Three replicates for each condition were tested.

**Supplementary Data 5. Results of ORFeome screen.** Primary data from analysis pipeline for each interaction in the chaperone screen data set. Log2FC = Log2 fold change, FDR = False Discovery Rate.

**Supplementary Data 6. Raw count data for ORFeome screen.** Count matrix for all barcoded yeast strains across each well tested for the ORFeome screen.

**Supplementary Data 7. Results of secondary validation of called hits and suspected interactions from the orfeome screen.** Three replicates for each condition were tested.

**Supplementary Data 8. RNA-seq results of FUS or TDP-43 overexpression. a.** Differential expression of chaperone genes upon FUS overexpression used in the generation of Supplementary Figure 16a. **b.** Differential expression of all genes upon FUS overexpression. **c.** Differential expression of chaperone genes upon TDP-43 overexpression used in the generation of Supplementary Figure 16a. **d.** Differential expression of all genes upon TDP-43 overexpression. Differential expression analysis was performed using the limma package in R. For each gene, log2 fold changes (logFC) and average log2 expression values (AveExpr) were calculated using topData(). Moderated t-statistics and associated p-values were computed, and multiple testing correction was applied using the Benjamini-Hochberg method to control the false discovery rate (FDR). Adjusted p-values (q-values) reflect the expected proportion of false positives among genes deemed significantly differentially expressed.

**Supplementary Data 9. Raw data from DNAJB6 and FUS co-localization studies, along with simulated data.**
