# Supplementary material for: Multiplex neurodegeneration proteotoxicity platform reveals DNAJB6 promotes non-toxic FUS condensate gelation and inhibits neurotoxicity
Source: Nat Commun. 2025 Nov 21;16:10285. doi: 10.1038/s41467-025-65178-0 (PMC12638947; doi:10.1038/s41467-025-65178-0)
Supplement: Supplementary file 12 — Reporting Summary [file 41467_2025_65178_MOESM12_ESM.pdf]

nature portfolio

Corresponding author(s): Alejandro Chavez
Peter St. George-Hyslop

Last updated by author(s): 08/29/2025

Reporting Summary

Nature Portfolio wishes to improve the reproducibility of the work that we publish. This form provides structure for consistency and transparency in reporting. For further information on Nature Portfolio policies, see our Editorial Policies and the Editorial Policy Checklist.

Statistics

For all statistical analyses, confirm that the following items are present in the figure legend, table legend, main text, or Methods section.

n/a	Confirmed	
☐	☒	The exact sample size (*n*) for each experimental group/condition, given as a discrete number and unit of measurement
☐	☒	A statement on whether measurements were taken from distinct samples or whether the same sample was measured repeatedly
☐	☒	The statistical test(s) used AND whether they are one- or two-sided<br>*Only common tests should be described solely by name; describe more complex techniques in the Methods section.*
☐	☒	A description of all covariates tested
☐	☒	A description of any assumptions or corrections, such as tests of normality and adjustment for multiple comparisons
☐	☒	A full description of the statistical parameters including central tendency (e.g. means) or other basic estimates (e.g. regression coefficient) AND variation (e.g. standard deviation) or associated estimates of uncertainty (e.g. confidence intervals)
☐	☒	For null hypothesis testing, the test statistic (e.g. *F*, *t*, *r*) with confidence intervals, effect sizes, degrees of freedom and *P* value noted<br>*Give P values as exact values whenever suitable.*
☒	☐	For Bayesian analysis, information on the choice of priors and Markov chain Monte Carlo settings
☒	☐	For hierarchical and complex designs, identification of the appropriate level for tests and full reporting of outcomes
☒	☐	Estimates of effect sizes (e.g. Cohen's *d*, Pearson's *r*), indicating how they were calculated

Our web collection on statistics for biologists contains articles on many of the points above.

Software and code

Policy information about availability of computer code

Data collection	Fabrication of Microfluidic Devices: Microfluidic devices were fabricated using a two-step photolithographic process with MicroChem's 3025 and SU-8 3050 photoresists. PDMS was used for device casting, and the PDMS chips were activated with O2 plasma.<br>Microfluidic Spray Deposition: Samples were introduced into the device using a syringe pump (Harvard Apparatus). Deposition was conducted for a maximum of 10 seconds on relevant surfaces (e.g., ZnSe crystals, FTIR prism).<br>Atomic Force Microscopy: Nanomechanical characterization was performed using a Bruker MultiMode 8 AFM system with ScanAsyst Fluid probes in force-volume or quantitative nanomechanical mapping mode. Images were acquired at scan rates of 0.3-0.5 Hz.<br>Fourier Transform Infrared Spectroscopy: FTIR measurements were performed using a Bruker Vertex 70 FTIR spectrometer with a DiamondATR unit and DLaTGS detector. Each spectrum was acquired as an average of 256 scans.<br>Infrared Nanospectroscopy (AFM-IR): Nanoscale infrared spectroscopy was performed using a Bruker nanoIR3 platform. AFM images were obtained with a silicon gold-coated probe in contact mode at a resolution of 500x100 pixels.<br>Granule Immunofluorescence Imaging: Airyscan experiments were conducted on an inverted Zeiss LSM 980 microscope with an Airyscan2 module, using standard laser lines for excitation. Multi-color Z-stack images were acquired.<br>Granule Detection and Colocalization: Granules were detected using a custom script in Fiji (ImageJ) with the Difference of Gaussians (DoG) detector from ImgLib2. Images were processed and analyzed to detect FUS and DNAJB6 colocalization.<br>Colocalization Simulation and Statistical Analysis: Colocalization was simulated by generating random positions of FUS and DNAJB6 signals within ROI masks using a custom script in MATLAB (R2021b).<br>Analysis of RNA Sequencing: Sequencing was performed on an Illumina NextSeq 550 machine, allocating ~30 million reads per replicate.
Data analysis	Atomic Force Microscopy: Force-distance curves were analyzed using Nanoscope Analysis Software Version 2.0 (Bruker).<br>Fourier Transform Infrared Spectroscopy: Spectral analysis was performed using OriginPro 2025 (OriginLab).

Infrared Nanospectroscopy (AFM-IR): Data were analyzed using SPIP Software Version 6.7.3 and OriginPRO 2025 (OriginLab).
Granule Immunofluorescence Imaging: Image processing was done using Zen Blue 3.10 (Zeiss).
Granule Detection and Colocalization: Statistical analysis of granules and colocalization was performed using R Statistical Software (v4.4).
Colocalization Simulation and Statistical Analysis: The overlap rate was analyzed using a right-tailed two-sample t-test, comparing experimental and simulated colocalization.
RNA Harvesting and Sequencing: Sequencing data were aligned to the hg19 genome using HISAT2 Version 2.0, and differential expression was calculated using limma.
Analysis of RNA Sequencing: Differential expression was calculated using limma.
Analysis of Multiplexed Screening: Raw reads were trimmed and assigned to wells using Illumina indexes and internal column designating indexes. Barcode sequences were aligned using bowtie2. Statistical analysis was performed using custom scripts in R (Version 4.0.2), including the calculation of CPM normalization and variance modeling.

For manuscripts utilizing custom algorithms or software that are central to the research but not yet described in published literature, software must be made available to editors and reviewers. We strongly encourage code deposition in a community repository (e.g. GitHub). See the Nature Portfolio guidelines for submitting code & software for further information.

Data

Policy information about availability of data

All manuscripts must include a data availability statement. This statement should provide the following information, where applicable:

- Accession codes, unique identifiers, or web links for publicly available datasets
- A description of any restrictions on data availability
- For clinical datasets or third party data, please ensure that the statement adheres to our policy

All reagents generated in this study will be deposited to Addgene. Raw sequencing reads from the chaperone screen have been uploaded to the NCBI SRA under BioProject PRJNA769721 (SUB10508463). Raw sequencing reads from the orfeome screen have been uploaded to the NCBI SRA under BioProject PRJNA769721 (SUB10508562). Raw sequencing reads from the deep mutational scanning have been uploaded to the NCBI SRA under BioProject PRJNA769721 (SUB10503160). Raw sequencing reads from the RNA-seq have been uploaded to the NCBI SRA under BioProject PRJNA769721 (SUB10426285). Hyperlink: NCBI SRA PRJNA769721 [https://www.ncbi.nlm.nih.gov/bioproject/?term=PRJNA769721]. Uncropped blots are provided as supplementary figures. The source data underlying all graphs presented within the main text and supplementary figures are provided as a Source Data File.

Research involving human participants, their data, or biological material

Policy information about studies with human participants or human data. See also policy information about sex, gender (identity/presentation), and sexual orientation and race, ethnicity and racism.

Reporting on sex and gender	N/A
Reporting on race, ethnicity, or other socially relevant groupings	N/A
Population characteristics	N/A
Recruitment	N/A
Ethics oversight	N/A

Note that full information on the approval of the study protocol must also be provided in the manuscript.

Field-specific reporting

Please select the one below that is the best fit for your research. If you are not sure, read the appropriate sections before making your selection.

- [x] Life sciences
- [ ] Behavioural & social sciences
- [ ] Ecological, evolutionary & environmental sciences

For a reference copy of the document with all sections, see nature.com/documents/nr-reporting-summary-flat.pdf

Life sciences study design

All studies must disclose on these points even when the disclosure is negative.

Sample size	Sample sizes were chosen based on known properties of each assay with regards to variance, formal power calculations were not performed
Data exclusions	No data excluded from analyses
Replication	All attempts at replication were successful
Randomization	Mice were randomly assigned after being genotyped to treatments
Blinding	Investigators were blind to animal treatment condition when assaying their histology.

Reporting for specific materials, systems and methods

We require information from authors about some types of materials, experimental systems and methods used in many studies. Here, indicate whether each material, system or method listed is relevant to your study. If you are not sure if a list item applies to your research, read the appropriate section before selecting a response.

Materials & experimental systems

n/a	Involved in the study
☐	☒ Antibodies
☐	☒ Eukaryotic cell lines
☒	☐ Palaeontology and archaeology
☐	☒ Animals and other organisms
☒	☐ Clinical data
☒	☐ Dual use research of concern
☒	☐ Plants

Methods

n/a	Involved in the study
☒	☐ ChIP-seq
☒	☐ Flow cytometry
☒	☐ MRI-based neuroimaging

Antibodies

Antibodies used	TDP-43 was detected with a polyclonal rabbit antibody at a 1:2,500 dilution (Proteintech 10782-2-AP). FUS was detected with a polyclonal rabbit antibody at a 1:2,500 dilution (Proteintech 11570-1-AP). hnRNPA1 was detected with a polyclonal rabbit antibody at a 1:5,000 dilution (Proteintech 11176-1-AP). DNAJB6 was detected with a monoclonal mouse antibody at a 1:2,500 dilution (Proteintech 66587-1-Ig). ChAT was detected with a polyclonal rabbit antibody at a 1:250 dilution (Millipore-Sigma AB144P). Iba1 was detected with a polyclonal rabbit antibody at a 1:5,000 dilution (Wako 019-19741). hDNAJB6 was detected with a monoclonal mouse antibody at a 1:1,000 dilution (Invitrogen MA5-27342). A goat anti-rabbit HRP conjugated antibody was used at a 1:50,000 dilution (Invitrogen G21234). A goat anti-mouse HRP conjugated antibody was used at a 1:10,000 dilution (Invitrogen 31430).
Validation	Antibodies were all validated by manufacturers.

Eukaryotic cell lines

Policy information about cell lines and Sex and Gender in Research

Cell line source(s)	human iPSC lines (CS29iALS-C9n1.ISOxx, Cedars Sinai; NSB3234, RUCDR)
Authentication	None of the cell lines were authenticated
Mycoplasma contamination	All cell lines tested negative for mycoplasma contamination
Commonly misidentified lines (See ICLAC register)	*Name any commonly misidentified cell lines used in the study and provide a rationale for their use.*

Animals and other research organisms

Policy information about studies involving animals; ARRIVE guidelines recommended for reporting animal research, and Sex and Gender in Research

Laboratory animals	C57Bl/6J (000664) and ChAT-CreΔneo (031661) mouse lines were obtained from Jackson Laboratory. Mice underwent neonatal injection and harvested at 6 months of age.
Wild animals	N/A
Reporting on sex	Sex was not considered in the study design and this information has not been collected.
Field-collected samples	N/A
Ethics oversight	All experiments involving live animals were approved by the Institutional Animal Care and Use Committee at Columbia University Irving Medical Center.

Note that full information on the approval of the study protocol must also be provided in the manuscript.

Plants

Seed stocks	N/A
Novel plant genotypes	N/A
Authentication	N/A
